# Synthesis of SF_5_-containing benzisoxazoles, quinolines, and quinazolines by the Davis reaction of nitro-(pentafluorosulfanyl)benzenes

**DOI:** 10.3762/bjoc.9.43

**Published:** 2013-02-21

**Authors:** Petr Beier, Tereza Pastýříková

**Affiliations:** 1 Institute of Organic Chemistry and Biochemistry, Academy of Sciences of the Czech Republic, Flemingovo nám. 2, 166 10 Prague, Czech Republic

**Keywords:** benzisoxazoles, pentafluorosulfanyl group, quinazolines, quinolines, sulfurpentafluorides

## Abstract

*Meta-* or *para-*nitro-(pentafluorosulfonyl)benzenes underwent the Davis reaction with arylacetonitriles to provide the SF_5_-containing benzisoxazoles. Good yields were obtained with arylacetonitriles containing the electron-neutral or electron-donor group, while those with the electron-acceptor group were found to be unreactive. Reductions of the benzisoxazoles gave *ortho*-aminobenzophenones in high yields. Their synthetic utility was demonstrated by condensation reactions with carbonyl compounds or amines to provide SF_5_-containing quinolines and quinazolines, respectively.

## Introduction

**Scheme 1:**
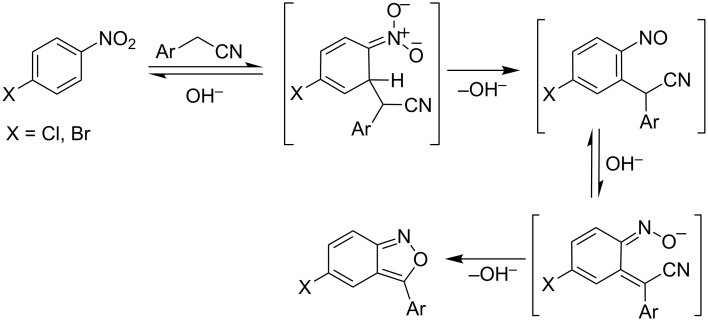
Proposed mechanism of the Davis reaction giving benzisoxazoles.

Reactions of nitroarenes with nucleophiles have been the focus of many investigations and represent processes that provide a range of useful products. These reactions can proceed in several ways: (a) *ipso* substitution of the nitro group of nitroarenes; (b) substitution of a halogen in halogen-substituted nitroarenes; and (c) substitution of hydrogen in *ortho-* or *para-*positions to the nitro group through vicarious or oxidative nucleophilic substitutions. Mechanistic studies have revealed that in all of these reaction pathways, the primary process is the reversible addition of nucleophiles to the ring carbon atoms bearing hydrogen and the formation of anionic σ^H^ adducts [1–6]. One example of such a reaction is the formation of benzisoxazoles (anthraniles) from substituted nitrobenzenes and arylacetonitriles in the presence of hydroxide in alcoholic solvent. This reaction was first reported by Davis and Pizzini [7] and probably proceeds through the formation of σ^H^ adducts that give nitroso compounds, which upon deprotonation enter an intramolecular addition–elimination process as shown in Scheme 1. Typical reaction conditions are an excess of alkali metal hydroxide in a low-boiling-point alcohol at ambient temperature [8–10].

**Figure 1:**
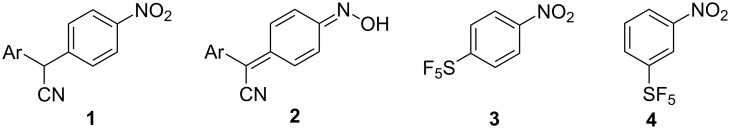
Substitution products **1**, oximes **2** and nitro-(pentafluorosulfanyl)benzenes **3** and **4**.

When the reaction was performed in pyridine, products **1** (Figure 1) of the nucleophilic substitution of halogen were formed. Reactions of *para-*nitroanisole with arylacetonitriles (KOH/MeOH) failed to provide the benzisoxazoles [7], and also dinitrobenzenes were found to be problematic [9]. The reactions of nitrobenzene with arylacetonitriles gave oximes **2** (Figure 1) [11].

Pentafluorosulfanyl-containing compounds (first synthesized by Sheppard in 1960 [12–13]) are relatively rare, and their chemistry is underdeveloped. These derivatives are promising for various applications due to an unusual combination of the properties of the SF_5_ group, such as high lipophilicity, with strong electron-acceptor character. One important property of the SF_5_ group is its high thermal and chemical stability [14–17]. The main reason that prevents further development of SF_5_ organics is their limited availability. In SF_5_-aromatics, *para*- and *meta*-nitro-(pentafluorosulfanyl)benzenes (**3** and **4**) (Figure 1) are available by the direct fluorination of the corresponding bis(nitrophenyl)disulfides [18–20], and several other SF_5_-benzenes are available through Umemoto’s two-step procedure starting from diaryldisulfides or mercaptoaromatics [21]. We have recently reported S_N_Ar reactions of the nitro group in compounds **3** and **4** with alkoxides and thiolates [22], vicarious nucleophilic substitution (VNS) of the hydrogen with carbon [23–24], oxygen [25] and nitrogen [26] nucleophiles, and oxidative nucleophilic substitution of hydrogen (ONSH) with Grignard and organolithium reagents [27]. This chemistry significantly expanded the range of available SF_5_-benzene derivatives. The synthetic chemistry and biological activity of pentafluorosulfanyl organic molecules has been recently reviewed [28].

**Scheme 2:**
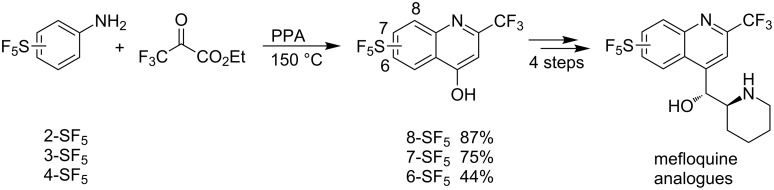
Synthesis of SF_5_-substituted quinolines and mefloquine analogues by Wipf and co-workers [29–30].

In this paper, we report our results on benzisoxazole formation from nitrobenzenes **3** and **4** and further transformations to SF_5_-containing aminobenzophenones, quinolines and quinazolines. Of these compound types, only some SF_5_-substituted quinolines are known. Wipf and co-workers have recently reported the synthesis of some SF_5_-substituted quinolines as intermediates towards analogues of the antimalarial agent mefloquine and found that some analogues had improved activity and selectivity against malaria parasites (Scheme 2) [29–30].

## Results and Discussion

We started the investigations by the reaction of *para-*nitro-(pentafluorosulfanyl)benzene (**3**) with phenylacetonitrile (**6a**) using excess sodium hydroxide in ethanol. The presence of the strongly electron-withdrawing SF_5_ group on nitrobenzene **3** should be beneficial for the Davis reaction since nitrobenzenes with electron-donor groups were found to be unreactive [7]. Addition of 1.5 equiv of **6a** and **3** to a solution of 10 equiv NaOH in ethanol produced a deep red–brown reaction mixture with the formation of a brown precipitate after a few minutes of stirring. After a further 15–30 minutes the precipitate dissolved, and after one hour the product **7a** was isolated in 66% yield (Table 1, entry 1). The benzisoxazole structure of **7a** was confirmed by spectroscopic methods. The reaction also worked in similar yields by using KOH in methanol, and no improvements were observed when the reaction time, temperature and amount of base and **6** were varied. Next, an investigation of the scope of the reaction was carried out. The presence of electron-donor groups on the benzene ring of **6** gave good yields of benzisoxazoles **7**; however, the reaction with (4-chlorophenyl)acetonitrile (**6b**) provided only traces of **7b** (no improvement of the yield was observed by increasing the amount of **6b** or using a longer reaction time).

**Table 1:** Synthesis of SF_5_-containing benzisoxazoles **7**–**9**.

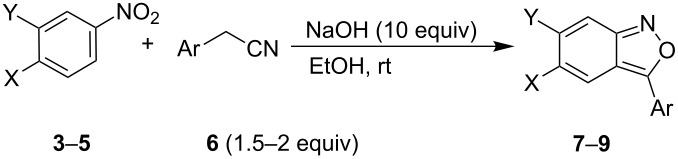						

Entry	**3–5**	X	Y	**6**, Ar	Time (h)	**7–9**, Yield (%)

1	**3**	SF_5_	H	**6a**, Ph	1	**7a**, 66
2	**3**	SF_5_	H	**6b**, 4-ClC_6_H_4_	48	**7b**, traces
3	**3**	SF_5_	H	**6c**, 3-MeOC_6_H_4_	1	**7c**, 65
4	**3**	SF_5_	H	**6d**, 3,4-(MeO)_2_C_6_H_3_	1	**7d**, 50
5	**3**	SF_5_	H	**6e**, 3,4,5-(MeO)_3_C_6_H_2_	1	**7e**, 54
6	**3**	SF_5_	H	**6f**, 4-PhC_6_H_4_	1	**7f**, 83
7	**4**	H	SF_5_	**6a**, Ph	1	**8a**, 83
8	**4**	H	SF_5_	**6b**, 4-ClC_6_H_4_	48	**8b**, traces
9	**5**	H	CF_3_	**6b**, 4-ClC_6_H_4_	1	**9b**, 58
10	**4**	H	SF_5_	**6c**, 3-MeOC_6_H_4_	2	**8c**, 59
11	**4**	H	SF_5_	**6d**, 3,4-(MeO)_2_C_6_H_3_	1.5	**8d**, 55
12	**4**	H	SF_5_	**6e**, 3,4,5-(MeO)_3_C_6_H_2_	1	**8e**, 57
13	**4**	H	SF_5_	**6g**, 3-IC_6_H_4_	48	**8g**, 0
14	**4**	H	SF_5_	**6h**, 4-NO_2_C_6_H_4_	168	**8h**, 0
15	**4**	H	SF_5_	**6i**, 2-F-6-ClC_6_H_4_	120	**8i**, 0

Reactions with the *meta-*nitro-(pentafluorosulfanyl)benzene (**4**) proceeded well with electron-neutral- or electron-donor-substituted phenylacetonitriles. With electron-acceptor-substituted phenylacetonitriles, the reactions failed. On the other hand, the reaction with 1-nitro-3-trifluoromethylbenzene (**5**) provided the benzisoxazole product in good yield. This observation is surprising given the fact that the CF_3_ and SF_5_ groups have similar group electronegativities [14], and the steric difference should not play a role here. These results indicate that the reaction scope shows limitations and the electronic properties of the starting substrates have to be finely tuned for efficient reaction.

The benzisoxazoles **7** and **8** were reduced to *ortho*-aminobenzophenones **10** and **11** in excellent yields by using iron powder in aqueous acetic acid according to the literature procedure (Table 2) [31].

**Table 2:** Synthesis of *ortho*-aminobenzophenones **10** and **11**.

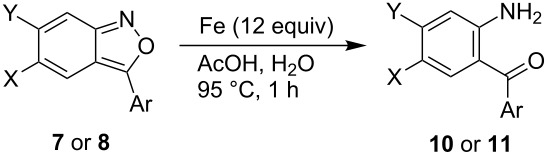					

Entry	**7** or **8**	X	Y	Ar	**10** or **11**, Yield (%)

1	**7a**	SF_5_	H	Ph	**10a**, 98
2	**7f**	SF_5_	H	4-PhC_6_H_4_	**10f**, 98
3	**8a**	H	SF_5_	Ph	**11a**, 93
4	**8d**	H	SF_5_	3,4-(MeO)_2_C_6_H_3_	**11d**, 98
5	**8e**	H	SF_5_	3,4,5-(MeO)_3_C_6_H_2_	**11e**, 98

**Scheme 3:**
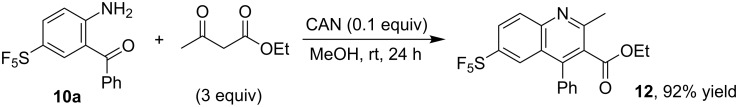
Synthesis of quinoline **12**.

Aminoketones **10** and **11** were investigated as starting substrates in the synthesis of various nitrogen heterocycles by condensation reactions. Several reliable synthetic methods giving quinolones [32–33] or quinazolines [34–35] have been reported in the literature. One potential problem of this approach is the reduced nitrogen nucleophilicity by the strongly electron-withdrawing SF_5_ group, especially for compounds **10** where the SF_5_ group is in conjugation with the amino group. The reduced reactivity was indeed observed compared to unsubstituted *ortho*-aminoacetophenone, but it could be overcome by using an excess of the condensation reagent and longer reaction times. Quinoline **12** was prepared in high yield by the Friedländer annulation reaction of **10a** with excess ethyl acetoacetate in the presence of catalytic CAN using modified literature conditions (Scheme 3) [32].

**Scheme 4:**
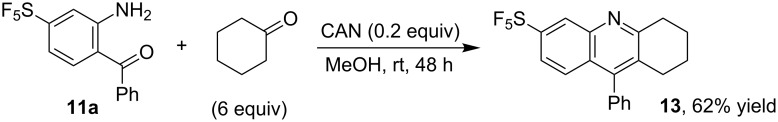
Synthesis of quinoline **13**.

Similarly, aminoketone **11a** was condensed in good yield with cyclohexanone to provide quinoline **13** in good yield (Scheme 4).

**Scheme 5:**
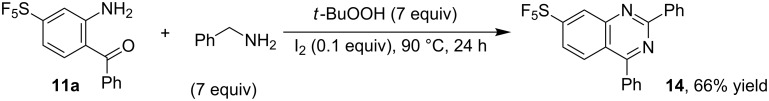
Synthesis of quinazoline **14**.

Finally, quinazoline **14** was synthesized by the reaction of aminoketone **11a** with benzylamine in the presence of *t*-BuOOH and catalytic iodine according to the literature conditions (Scheme 5) [34]. However, the analogous reaction with ketone **11e** was found to be too slow (36% conversion after 26 h as judged by GCMS analysis) to be synthetically useful. This reduced reactivity of **11e** compared to **11a** is due to the relatively low electrophilicity of the carbonyl group of **11e**.

## Conclusion

In summary, reactions of *meta-* or *para-*nitro-(pentafluorosulfonyl)benzenes with arylacetonitriles in the presence of NaOH in ethanol gave the SF_5_-containing benzisoxazoles in good to high yields. These reactions are limited to arylacetonitriles containing electron-neutral or electron-donor groups. Reductions of the benzisoxazoles with iron powder in acetic acid provided high yields of SF_5_-containing *ortho*-aminobenzophenones, which underwent condensation reactions to quinolines or quinazolines. This methodology provides straightforward access to new SF_5_-substituted aromatic and nitrogen-containing heteroaromatic compounds.

## Supporting Information

File 1 Experimental details, characterization data, and copies of NMR spectra for all new compounds.
