# Supplementary material for: Synthesis of SF5-containing benzisoxazoles, quinolines, and quinazolines by the Davis reaction of nitro-(pentafluorosulfanyl)benzenes
Source: Beilstein J Org Chem. 2013 Feb 21;9:411–6. doi: 10.3762/bjoc.9.43 (PMC3596107; doi:10.3762/bjoc.9.43)
Supplement: File 1 — Experimental details, characterization data, and copies of NMR spectra for all new compounds. [file Beilstein_J_Org_Chem-09-411-s001.pdf]

Supporting Information

for

Synthesis of SF_5-containing benzisoxazoles, quinolines, and quinazolines by the Davis reaction of nitro-(pentafluorosulfanyl)benzenes

Petr Beier* and Tereza Pastýříková

Address: Institute of Organic Chemistry and Biochemistry, Academy of Sciences of the Czech Republic, Flemingovo nám. 2, 166 10 Prague, Czech Republic

Email: Petr Beier - beier@uochb.cas.cz

* Corresponding author

Experimental details, characterization data, and copies of NMR spectra for all new compounds

1. General experimental procedures

Infrared spectra were measured on Bruker Equinox 55 FTIR instrument. UV–vis spectra were measured on a Varian Cary 5000 instrument. NMR spectra were recorded at rt on Bruker Avance 400 or 500 MHz instruments. Chemical shifts (δ) are reported in ppm relative to Me_4Si (0 ppm, for ^1H NMR), residual $CHCl_3$ (7.26 ppm for ^1H NMR), $CDCl_3$ (77.0 ppm for ^{13}C NMR), and internal $CFCl_3$ (0 ppm for ^{19}F NMR). GC–MS spectra were recorded on an Agilent 7890A gas chromatograph coupled with a 5975C quadrupole mass-selective electron impact (EI) detector (70 eV). High-resolution mass spectra (HRMS) were recorded on an Agilent 7890A gas chromatograph coupled with a Waters GCT Premier orthogonal acceleration time-of-flight detector using electron impact (EI) or chemical ionizations (CI) or on a LTQ Orbitrap XL instrument using electrospray ionization (ESI). PE refers to petroleum ether bp 40–60 °C.

2. General procedure for the synthesis of benzisoxazoles 7–9

Powdered NaOH (0.8 g, 20 mmol, 10 equiv) was stirred with ethanol (10 mL) at rt for 20 min. A mixture of **3** or **4** (500 mg, 2 mmol) and arylacetonitrile **6** (3–4 mmol, 1.5–2 equiv) was added, and the mixture was stirred in a closed reaction flask at rt for the given time (1–2 h) and then poured into water (70 mL). The crude product was extracted into EtOAc (3 × 30 mL). The combined organic phase was washed with saturated NH_4Cl (20 mL), dried and the solvent was removed under reduced pressure. Flash chromatography using silica gel (EtOAc–PE) provided pure products.

2.1. 3-Phenyl-5-(pentafluorosulfanyl)benzo[*c*]isoxazole (7a)

A white solid (66% yield); mp 129–130 °C; R_f 0.40 (EtOAc–PE, 4:96); IR (film) ν_{max} 3128, 3090, 3050, 1631, 1547, 1521, 1495, 1466, 1447, 1368, 1144, 1064, 935, 839; ^{1}H NMR (500 MHz, $CDCl_3$) δ_H 7.56–7.64 (m, 3H), 7.65 (dd, 1H, $^3J_{HH}$ = 9.8 Hz, $^4J_{HH}$ = 1.8 Hz), 7.67 (br d, 1H, $^3J_{HH}$ = 9.8 Hz), 7.98 (m, 2H), 8.31 (br dd, 1H, $^4J_{HH}$ = 1.8 Hz, $^4J_{HF}$ = 0.8 Hz); ^{13}C NMR (125.7 MHz, $CDCl_3$) δ_C 112.4, 116.3, 121.3 (quint., $^3J_{CF}$ = 5.3 Hz), 127.0, 127.2, 127.5 (quint., $^3J_{CF}$ = 4.1 Hz), 129.6, 131.5, 150.4 (quint., $^2J_{CF}$ = 18.1 Hz), 156.4, 168.6; ^{19}F NMR (470.3 MHz, $CDCl_3$) δ_F 62.8 (d, 4F, $^2J_{FF}$ = 150.4 Hz), 83.6 (quint., 1F, $^2J_{FF}$ = 150.4 Hz); MS (EI) *m*/*z* (rel. int.) 322 (32), 321 (100) $[M]^+$, 213 (55), 194 (24), 185 (35), 184 (21), 166 (55), 140 (21), 139 (32), 105 (18), 77 (70), 51 (19); HRMS (EI) *m*/*z* calcd for $C_{13}H_8F_5NOS$ $[M]^+$ 321.0247, found 321.0244.

2.2. 5-(Pentafluorosulfanyl)-3-(3-methoxyphenyl)benzo[*c*]isoxazole (7c)

A pale yellow solid (65% yield); mp 108.4–109.8 °C; R_f 0.50 (EtOAc–PE, 10:90); IR (film) ν_{max} 3007, 2943, 2840, 1635, 1602, 1583, 1552, 1523, 1495, 1468, 1436, 1367, 1250, 1047, 935, 843, 824; ^{1}H NMR (500 MHz, $CDCl_3$) δ_H 3.91 (s, 3H), 7.10 (ddd, 1H, $^3J_{HH}$ = 7.8 Hz, $^4J_{HH}$ = 2.6, 1.4 Hz), 7.48 (ddd, 1H, $^4J_{HH}$ = 2.6, 1.6 Hz, $^5J_{HH}$ = 0.5 Hz), 7.50 (ddd, 1H, $^3J_{HH}$ = 7.8, 7.7 Hz, $^5J_{HH}$ = 0.5 Hz), 7.53 (ddd, 1H, $^3J_{HH}$ = 7.7 Hz, $^4J_{HH}$ = 1.6, 1.4 Hz), 7.64 (dd, 1H, $^3J_{HH}$ = 9.8 Hz, $^4J_{HH}$ = 1.8 Hz), 7.66 (br d, 1H, $^3J_{HH}$ = 9.8 Hz), 8.28 (dd, 1H, $^4J_{HH}$ = 1.8 Hz, $^5J_{HH}$ = 0.9 Hz); ^{13}C NMR (125.7 MHz, $CDCl_3$) δ_C 55.5, 112.2, 112.4, 116.2, 117.3, 119.3, 121.3 (quint., $^3J_{CF}$ = 5.2 Hz), 127.4 (quint., $^3J_{CF}$ = 4.1 Hz), 128.1, 130.7, 150.4 (quint., $^2J_{CF}$ = 18.3 Hz), 156.3, 160.2, 168.4; ^{19}F NMR (470.3 MHz, $CDCl_3$) δ_F 62.8 (d, 4F, $^2J_{FF}$ = 150.4 Hz), 83.6 (quint., 1F, $^2J_{FF}$ = 150.4 Hz); MS (ESI) *m*/*z* (rel. int.) 352 (32) $[M + H]^+$, 332 (100), 224 (90); HRMS (ESI) *m*/*z* calcd for $C_{14}H_{11}F_5NO_2S$ $[M + H]^+$ 352.04252, found 352.05233.

2.3. 5-(Pentafluorosulfanyl)-3-(3,4-dimethoxyphenyl)benzo[*c*]isoxazole (7d)

A yellow solid (50% yield); mp 117–118 °C; R_f 0.34 (EtOAc–PE, 20:80); IR (film) ν_{max} 3089, 3006, 2963, 2940, 2841, 1634, 1601, 1587, 1553, 1512, 1467, 1436, 1370, 1265, 1240, 1142, 1134, 1065, 1024, 933, 843; ^{1}H NMR (500 MHz, $CDCl_3$) δ_H 4.00 (s, 3H), 4.02 (s, 3H), 7.08 (d, 1H, $^3J_{HH}$ = 8.4 Hz), 7.51 (d, 1H, $^4J_{HH}$ = 2.0 Hz), 7.58 (dd, 1H, $^3J_{HH}$ = 8.4 Hz, $^4J_{HH}$ = 2.0 Hz), 7.64 (d, 2H, J_{HH} = 1.4 Hz), 8.27 (t, 1H, J_{HH} = 1.4 Hz); ^{13}C NMR (125.7 MHz, $CDCl_3$) δ_C 56.10, 56.14, 109.6, 111.6, 111.7, 116.0, 120.0, 120.5, 121.5 (quint., $^3J_{CF}$ = 5.2 Hz), 127.4 (quint., $^3J_{CF}$ = 4.1 Hz), 149.8, 150.0 (quint., $^2J_{CF}$ = 18.7 Hz), 152.0, 156.4, 168.7; ^{19}F NMR (470.3 MHz, $CDCl_3$) δ_F 62.9 (d, 4F, $^2J_{FF}$ = 150.4 Hz), 83.9 (quint., 1F, $^2J_{FF}$ = 150.4 Hz); MS (ESI) *m*/*z* (rel. int.) 404 (70) $[M + Na]^+$, 382 (43) $[M + H]^+$, 254 (45); HRMS (ESI) *m*/*z* calcd for $C_{15}H_{13}F_5NO_3S$ $[M + H]^+$ 382.05308, found 382.05287.

2.4. 5-(Pentafluorosulfanyl)-3-(3,4,5-trimethoxyphenyl)benzo[*c*]isoxazole (7e)

A pale yellow solid (54% yield); mp 138–139 °C; R_f 0.44 (EtOAc–PE, 15:85); IR (film) ν_{max} 2942, 2836, 2362, 2337, 1633, 1585, 1552, 1503, 1467, 1418, 1377, 1240, 1128, 837, 824; ^{1}H NMR (500 MHz, $CDCl_3$) δ_H 3.97 (s, 3H), 4.00 (s, 6H), 7.14 (s, 2H), 7.62 (d, 2H, J_{HH} = 1.4 Hz), 8.23 (t, 1H, J_{HH} = 1.4 Hz); ^{13}C NMR (125.7 MHz, $CDCl_3$) δ_C 56.2, 60.8, 104.3, 111.9, 116.0, 121.1 (quint., $^3J_{CF}$ = 5.1 Hz), 122.0, 127.2 (quint., $^3J_{CF}$ = 3.8 Hz), 141.0, 150.1 (quint., $^2J_{CF}$ = 17.9 Hz), 153.9, 156.2, 168.4; ^{19}F NMR (470.3 MHz, $CDCl_3$) δ_F 62.8 (d, 4F, $^2J_{FF}$ = 150.3 Hz), 83.8 (quint., 1F, $^2J_{FF}$ = 150.3 Hz); MS (ESI) *m*/*z* (rel. int.) 434 (100) $[M + Na]^+$, 412 (8) $[M + H]^+$, 381 (20), 379 (17), 368 (12), 284 (10); HRMS (ESI) *m*/*z* calcd for $C_{16}H_{14}F_5NNaO_4S$ $[M + Na]^+$ 434.04559, found 434.04563.

2.5. 3-(Biphenyl-4-yl)-5-(pentafluorosulfanyl)benzo[*c*]isoxazole (7f)

A pale yellow solid (83% yield); mp 156–157 °C; R_f 0.44 (EtOAc–PE, 10:90); IR (film) ν_{max} 3060, 3033, 1665, 1634, 1606, 1582, 1564, 1537, 1513, 1489, 1465, 1409, 1370, 1063, 1007, 932, 840; ^{1}H NMR (500 MHz, $CDCl_3$) δ_H 7.39 (m, 1H), 7.46 (m, 2H), 7.61 (m, 2H), 7.62 (m, 2H), 7.76 (m, 2H), 7.99 (m, 2H), 8.30 (br s, 1H); ^{13}C NMR (125.7 MHz, $CDCl_3$) δ_C 112.3, 116.2, 121.3 (quint., $^3J_{CF}$ = 5.1 Hz), 125.7, 127.0, 127.2, 127.3 (quint., $^3J_{CF}$ = 3.9 Hz), 128.0, 128.3, 129.0, 139.3, 144.0, 150.3 (quint., $^2J_{CF}$ = 18.2 Hz), 156.3, 168.2; ^{19}F NMR (470.3 MHz, $CDCl_3$) δ_F 62.9 (d, 4F, $^2J_{FF}$ = 150.5 Hz), 83.8 (quint., 1F, $^2J_{FF}$ = 150.5 Hz); MS (EI) *m*/*z* (rel. int.) 399 (75), 398 (100), 397 (50) $[M]^+$, 322 (30), 181 (38), 153 (30), 152 (56); HRMS (EI) *m*/*z* calcd for $C_{19}H_{12}F_5NOS$ $[M]^+$ 397.0560, found 397.0566.

2.6. 3-Phenyl-6-(pentafluorosulfanyl)benzo[*c*]isoxazole (8a)

A white solid (83% yield); mp 120–122 °C; R_f 0.49 (EtOAc–PE, 5:95); IR (film) ν_{max} 3119, 3092, 3060, 1633, 1601, 1551, 1517, 1496, 1456, 1438, 842; ^{1}H NMR (500 MHz, $CDCl_3$) δ_H 7.35 (dd, 1H, $^3J_{HH}$ = 9.5 Hz, $^4J_{HH}$ = 1.8 Hz), 7.50–7.57 (m, 3H), 7.87 (dsex, 1H, $^3J_{HH}$ = 9.5 Hz, $^5J_{HH}$ = $^5J_{HF}$ = 0.9 Hz), 7.95 (m, 2H), 8.07 (dd, 1H, $^4J_{HH}$ = 1.8 Hz, $^5J_{HH}$ = 0.9 Hz); ^{13}C NMR (125.7 MHz, $CDCl_3$) δ_C 113.7, 115.6 (quint., $^3J_{CF}$ = 5.3 Hz), 121.0 (quint., $^3J_{CF}$ = 4.2 Hz), 121.9, 126.6, 127.3, 129.4, 131.1, 155.5 (quint., $^2J_{CF}$ = 18.1 Hz), 156.2, 166.1; ^{19}F NMR (470.3 MHz, $CDCl_3$) δ_F 61.6 (d, 4F, $^2J_{FF}$ = 150.2 Hz), 82.2 (quint., 1F, $^2J_{FF}$ = 150.2 Hz); MS (EI) *m*/*z* (rel. int.) 322 (19), 321 (100) $[M]^+$, 213 (25), 194 (19), 185 (18), 166 (31), 139 (19), 105 (10), 77 (38); HRMS (EI) *m*/*z* calcd for $C_{13}H_8F_5NOS$ $[M]^+$ 321.0247, found 321.0249.

2.7. 3-(4-Chlorophenyl)-6-(trifluoromethyl)benzo[*c*]isoxazole (9b)

A yellow solid (58% yield); mp 146–147 °C; R_f 0.56 (EtOAc–PE, 10:90); IR (film) ν_{max} 3118, 3097, 3056, 1648, 1595, 1578, 1523, 1499, 1461, 1438, 1398, 1347, 1271, 1171, 1153, 1128, 1096; ^{1}H NMR (400 MHz, $CDCl_3$) δ_H 7.20 (dd, 1H, $^3J_{HH}$ = 9.2 Hz, J_{HH} = 1.3 Hz), 7.52–7.56 (m, 2H), 7.89–7.96 (m, 4H); ^{13}C NMR (100 MHz, $CDCl_3$) δ_C 114.6 (q, $^3J_{CF}$ = 5.2 Hz), 120.5 (q, $^3J_{CF}$ = 2.7 Hz), 122.2, 123.3 (q, $^1J_{CF}$ = 272.9 Hz), 126.1, 127.8, 129.8, 133.0 (q, $^2J_{CF}$ = 32.7 Hz), 137.2, 156.6, 164.6; ^{19}F NMR (376 MHz, $CDCl_3$) δ_F –65.0 (s); MS (ESI) *m*/*z* (rel. int.) 300 (22), 299 (22), 298 (100) $[M + H]^+$, 297 (55), 288 (12), 280 (18), 278 (46); HRMS (ESI) *m*/*z* calcd for $C_{14}H_8ClF_3NO$ $[M + H]^+$ 298.02410, found 398.02403.

2.8. 6-(Pentafluorosulfanyl)-3-(3-methoxyphenyl)benzo[*c*]isoxazole (8c)

A pale yellow solid (59% yield); mp 88.0–89.4 °C; R_f 0.43 (EtOAc–PE, 10:90); IR (film) ν_{max} 3085, 3008, 2944, 2840, 1634, 1601, 1582, 1554, 1516, 1495, 1463, 1437, 1290, 1247, 1061, 1048, 843, 820; ^{1}H NMR (500 MHz, $CDCl_3$) δ_H 3.89 (s, 3H), 7.04 (ddd, 1H, $^3J_{HH}$ = 8.2 Hz, $^4J_{HH}$ = 2.6, 1.0 Hz), 7.34 (dd, 1H, $^3J_{HH}$ = 9.5 Hz, $^4J_{HH}$ = 1.8 Hz), 7.44 (dd, 1H, $^3J_{HH}$ = 8.2, 7.7 Hz), 7.45 (dd, 1H, $^4J_{HH}$ = 2.6, 1.6 Hz), 7.51 (ddd, 1H, $^3J_{HH}$ = 7.7 Hz, $^4J_{HH}$ = 1.6, 1.0 Hz), 7.85 (d, 1H, $^3J_{HH}$ = 9.5 Hz), 8.07 (dd, 1H, $^4J_{HH}$ = 1.8 Hz, $^5J_{HH}$ = 0.8 Hz); ^{13}C NMR (125.7 MHz, $CDCl_3$) δ_C 55.3, 111.8, 113.7, 115.5 (quint., $^3J_{CF}$ = 5.2 Hz), 116.8, 118.8, 121.0 (quint., $^3J_{CF}$ = 4.2 Hz), 121.9, 128.3, 130.5, 155.5 (quint., $^2J_{CF}$ = 17.9 Hz), 156.2, 160.1, 165.9; ^{19}F NMR (470.3 MHz, $CDCl_3$) δ_F 61.6 (d, 4F, $^2J_{FF}$ = 150.2 Hz), 82.2 (quint., 1F, $^2J_{FF}$ = 150.2 Hz); MS (ESI) *m*/*z* (rel. int.) 352 (100) $[M + H]^+$, 337 (20), 244 (50), 229 (13); HRMS (ESI) *m*/*z* calcd for $C_{14}H_{11}F_5NO_2S$ $[M + H]^+$ 352.04252, found 352.04251.

2.9. 6-(Pentafluorosulfanyl)-3-(3,4-dimethoxyphenyl)benzo[*c*]isoxazole (8d)

A yellow solid (55% yield); mp 128–129 °C; R_f 0.30 (EtOAc–PE, 20:80); IR (film) ν_{max} 3087, 3008, 2965, 2940, 2841, 1632, 1603, 1585, 1553, 1524, 1506, 1458, 1436, 1264, 1239, 1142, 1129, 1061, 1024, 916, 847; ^{1}H NMR (500 MHz, $CDCl_3$) δ_H 3.99 (s, 3H), 4.01 (s, 3H), 7.04 (d, 1H, $^3J_{HH}$ = 8.5 Hz), 7.35 (dd, 1H, $^3J_{HH}$ = 9.5 Hz, $^4J_{HH}$ = 1.8 Hz), 7.51 (d, 1H, $^4J_{HH}$ = 2.1 Hz), 7.59 (dd, 1H, $^3J_{HH}$ = 8.5 Hz, $^4J_{HH}$ = 2.1 Hz), 7.88 (dsex, 1H, $^3J_{HH}$ = 9.5 Hz, $^5J_{HH}$ = $^5J_{HF}$ = 0.8 Hz), 8.08 (dd, 1H, $^4J_{HH}$ = 1.8 Hz, $^5J_{HH}$ = 0.8 Hz); ^{13}C NMR (125.7 MHz, $CDCl_3$) δ_C 56.06, 56.1, 119.3, 111.5, 113.1, 115.4 (quint., $^3J_{CF}$ = 5.2 Hz), 120.1, 120.3, 120.6, 122.1, 149.7, 151.6, 155.6 (quint., $^2J_{CF}$ = 18.0 Hz), 156.4, 166.2; ^{19}F NMR (470.3 MHz, $CDCl_3$) δ_F 61.6 (d, 4F, $^2J_{FF}$ = 150.1 Hz), 82.2 (quint., 1F, $^2J_{FF}$ = 150.1 Hz); MS (ESI) *m*/*z* (rel. int.) 404 (100) $[M + Na]^+$, 382 (40) $[M + H]^+$, 254 (40); HRMS (ESI) *m*/*z* calcd for $C_{15}H_{13}F_5NO_3S$ $[M + H]^+$ 382.05308, found 382.05294.

2.10. 6-(Pentafluorosulfanyl)-3-(3,4,5-trimethoxyphenyl)benzo[*c*]isoxazole (8e)

A pale yellow solid (57% yield); mp 149.5–150.5 °C; R_f 0.15 (EtOAc–PE, 7:93); IR (film) ν_{max} 3110, 3082, 3063, 3004, 2946, 2839, 1633, 1586, 1553, 1501, 1460, 1428, 1400, 1334, 1301, 1276, 1242, 1134, 1127, 1060, 1001, 845, 826, 776; ^{1}H NMR (500 MHz, $CDCl_3$) δ_H 3.96 (s, 3H), 3.99 (s, 6H), 7.20 (s, 2H), 7.38 (dd, 1H, $^3J_{HH}$ = 9.5 Hz, $^4J_{HH}$ = 1.8 Hz), 7.87 (d, 1H, $^3J_{HH}$ = 9.5 Hz), 8.09 (dd, 1H, $^4J_{HH}$ = 1.8 Hz, $^5J_{HH}$ = 0.8 Hz); ^{13}C NMR (125.7 MHz, $CDCl_3$) δ_C 56.3, 61.0, 104.1, 113.4, 115.5 (quint., $^3J_{CF}$ = 5.1 Hz), 120.9 (quint., $^3J_{CF}$ = 4.1 Hz), 121.8, 122.5, 140.8, 153.9, 155.5 (quint., $^2J_{CF}$ = 18.5 Hz), 156.3, 166.0; ^{19}F NMR (470.3 MHz, $CDCl_3$) δ_F 61.6 (d, 4F, $^2J_{FF}$ = 150.2 Hz), 82.1 (quint., 1F, $^2J_{FF}$ = 150.2 Hz); MS (ESI) *m*/*z* (rel. int.) 434 (100) $[M + Na]^+$, 412 (25) $[M + H]^+$; HRMS (ESI) *m*/*z* calcd for $C_{16}H_{15}F_5NO_4S$ $[M + H]^+$ 412.06365, found 412.06369.

3. General procedure for the reduction of benzisoxazoles to *ortho*-aminobenzophenones 10 and 11

Benzisoxazole **7** or **8** (0.55 mmol) was dissolved in acetic acid (8.2 mL) and a suspension of iron powder (369 mg, 6.6 mmol, 12 equiv) in water (0.5 mL) was added. The mixture was heated at 95 °C for 1 h, then water (50 mL) was added and the product was extracted into EtOAc (3 × 20 mL). The combined organic phase was washed with brine (20 mL) and dried, and the solvent was removed under reduced pressure giving pure products **10** or **11**.

3.1. (2-Amino-5-(pentafluorosulfanyl)phenyl)(phenyl)methanone (10a)

A pale yellow oil (98% yield); R_f 0.25 (EtOAc–PE, 15:85); IR (film) ν_{max} 3473, 3351, 3100, 3081, 3064, 3033, 1638, 1618, 1587, 1549, 1483, 1308, 1261, 1179, 1106, 951, 840; ^{1}H NMR (500 MHz, $CDCl_3$) δ_H 6.51 (br s, 2H), 6.69 (br d, 1H, $^3J_{HH}$ = 9.2 Hz), 7.48 (m, 2H), 7.57 (m, 1H), 7.60 (dd, 1H, $^3J_{HH}$ = 9.2 Hz, $^4J_{HH}$ = 2.6 Hz), 7.64 (m, 2H), 7.90 (d, 1H, $^4J_{HH}$ = 2.6 Hz); ^{13}C NMR (125.7 MHz, $CDCl_3$) δ_C 115.8, 116.2, 128.4, 129.2, 131.1 (quint., $^3J_{CF}$ = 4.2 Hz), 132.0, 132.5 (quint., $^3J_{CF}$ = 4.6 Hz), 138.6, 141.3 (quint., $^2J_{CF}$ = 18.5 Hz), 152.4, 197.8; ^{19}F NMR (470.3 MHz, $CDCl_3$) δ_F 63.7 (d, 4F, $^2J_{FF}$ = 150.5 Hz), 86.3 (quint., 1F, $^2J_{FF}$ = 150.5 Hz); MS (EI) *m*/*z* (rel. int.) 324 (16), 323 (81) $[M]^+$, 322 (100), 246 (35), 214 (10), 105 (46), 77 (48); HRMS (EI) *m*/*z* calcd for $C_{13}H_{10}F_5NOS$ $[M]^+$ 323.0403, found 323.0394.

3.2. (2-Amino-5-(pentafluorosulfanyl)phenyl)(biphenyl-4-yl)-methanone (10f)

A pale yellow solid (98% yield); mp 144.0–146.0 °C; R_f 0.22 (EtOAc–PE, 15:85); IR (film) ν_{max} 3478, 3348, 1640, 1616, 1586, 1548, 1514, 1485, 1401, 1310, 1257, 1179, 1104, 947, 836, 811; ^{1}H NMR (500 MHz, $CDCl_3$) δ_H 6.72 (d, 1H, $^3J_{HH}$ = 9.1 Hz), 7.41 (m, 1H), 7.48 (m, 2H), 7.64 (dd, 1H, $^3J_{HH}$ = 9.1 Hz, $^4J_{HH}$ = 2.6 Hz), 7.66 (m, 2H), 7.72 (m, 2H), 7.74 (m, 2H), 7.97 (d, 1H, $^4J_{HH}$ = 2.6 Hz); ^{13}C NMR (125.7 MHz, $CDCl_3$) δ_C 116.1, 116.2, 127.1, 127.3, 128.2, 129.0, 130.0, 131.1 (quint., $^3J_{CF}$ = 4.1 Hz), 132.4 (quint., $^3J_{CF}$ = 4.5 Hz), 137.2, 139.8, 141.5 (quint., $^2J_{CF}$ = 18.1 Hz), 144.9, 152.3, 197.3; ^{19}F NMR (376 MHz, $CDCl_3$) δ_F 63.7 (d, 4F, $^2J_{FF}$ = 150.5 Hz), 86.2 (quint., 1F, $^2J_{FF}$ = 150.5 Hz); MS (EI) *m*/*z* (rel. int.) 400 (20), 399 (81) $[M]^+$, 398 (100), 322 (34), 181 (36), 153 (22), 152 (46); HRMS (ESI) *m*/*z* calcd for $C_{19}H_{15}F_5NOS$ $[M + H]^+$ 400.07890, found 400.07893.

3.3. (2-Amino-4-(pentafluorosulfanyl)phenyl)(phenyl)methanone (11a)

A yellow oil (93% yield); R_f 0.61 (EtOAc–PE, 20:80); IR (film) ν_{max} 3475, 3357, 3105, 3084, 3065, 3023, 1640, 1619, 1580, 1552, 1486, 1432, 1318, 1248, 894, 842, 807; ^{1}H NMR (500 MHz, $CDCl_3$) δ_H 6.28 (br s, 2H), 6.92 (dd, 1H, $^3J_{HH}$ = 8.8 Hz, $^4J_{HH}$ = 2.2 Hz), 7.14 (d, 1H, $^4J_{HH}$ = 2.2 Hz), 7.45 (m, 2H), 7.50 (br d, 1H, $^3J_{HH}$ = 8.8 Hz), 7.54 (m, 1H), 7.63 (m, 2H); ^{13}C NMR (125.7 MHz, $CDCl_3$) δ_C 112.1 (quint., $^3J_{CF}$ = 4.5 Hz), 114.5 (quint., $^3J_{CF}$ = 4.7 Hz), 119.4, 128.2, 129.1, 131.8, 134.5, 138.8, 150.5, 157.2 (quint., $^2J_{CF}$ = 17.5 Hz), 198.0; ^{19}F NMR (470.3 MHz, $CDCl_3$) δ_F 61.2 (d, 4F, $^2J_{FF}$ = 150.0 Hz), 83.2 (quint., 1F, $^2J_{FF}$ = 150.0 Hz); MS (EI) *m*/*z* (rel. int.) 324 (14), 323 (81) $[M]^+$, 322 (100), 246 (24), 214 (16), 105 (43), 77 (47); HRMS (EI) *m*/*z* calcd for $C_{13}H_{10}F_5NOS$ $[M]^+$ 323.0403, found 323.0408.

3.4. (2-Amino-4-(pentafluorosulfanyl)phenyl)(3,4-dimethoxyphenyl)methanone (11d)

A pale yellow solid (98% yield), mp 104.0–105.8 °C; R_f 0.11 (EtOAc–PE, 15:85); IR (film) ν_{max} 3465, 3361, 2938, 2841, 2362, 2337, 1635, 1615, 1581, 1513, 1488, 1465, 1417, 1306, 1262, 1229, 1128, 1024, 927, 843, 804; ^{1}H NMR (400 MHz, $CDCl_3$) δ_H 3.94 (s, 3H), 3.97 (s, 3H), 5.92 (br s, 2H), 6.91 (d, 1H, $^3J_{HH}$ = 8.4 Hz), 6.99 (dd, 1H, $^3J_{HH}$ = 8.7 Hz, $^4J_{HH}$ = 2.1 Hz), 7.14 (d, 1H, $^4J_{HH}$ = 2.1 Hz), 7.25–7.28 (m, 1H), 7.34 (d, 1H, $^4J_{HH}$ = 1.8 Hz), 7.54 (d, 1H, $^3J_{HH}$ = 8.7 Hz); ^{13}C NMR (100 MHz, $CDCl_3$) δ_C 56.0, 56.1, 109.9, 111.9, 112.5 (quint., $^3J_{CF}$ = 4.6 Hz), 114.4 (quint., $^3J_{CF}$ = 4.7 Hz), 120.8, 124.6, 131.2, 133.7, 149.0, 149.0, 149.8, 152.8, 157.0 (quint., $^2J_{CF}$ = 17.3 Hz), 196.2; ^{19}F NMR (3763 MHz, $CDCl_3$) δ_F 61.2 (d, 4F, $^2J_{FF}$ = 149.9 Hz), 83.2 (quint., 1F, $^2J_{FF}$ = 149.9 Hz); MS (EI) *m*/*z* (rel. int.) 384 (17), 383 (79) $[M]^+$, 382 (100), 369 (10), 368 (63), 366 (14), 336 (14), 352 (13), 165 (29), 138 (17); HRMS (ESI) *m*/*z* calcd for $C_{14}H_{14}F_5NNaO_3S$ $[M + Na]^+$ 406.05068, found 406.05066.

3.5. (2-Amino-4-(pentafluorosulfanyl)phenyl)(3,4,5-trimethoxyphenyl)methanone (11e)

A pale yellow solid (98% yield), mp 128.5–129.4 °C; R_f 0.16 (EtOAc–PE, 15:85); IR (film) ν_{max} 3464, 3356, 2943, 2840, 1640, 1617, 1583, 1550, 1504, 1488, 1465, 1415, 1333, 1230, 1129, 1002, 930, 845, 823, 805; ^{1}H NMR (500 MHz, $CDCl_3$) δ_H 3.89 (s, 6H), 3.94 (s, 3H), 6.18 (br s, 2H), 6.93 (s, 2H), 6.97 (dd, 1H, $^3J_{HH}$ = 8.8 Hz, $^4J_{HH}$ = 2.2 Hz), 7.17 (d, 1H, $^4J_{HH}$ = 2.2 Hz), 7.57 (br d, 1H, $^3J_{HH}$ = 8.8 Hz); ^{13}C NMR (125.7 MHz, $CDCl_3$) δ_C 56.2, 60.8, 106.8, 112.2 (quint., $^3J_{CF}$ = 4.4 Hz), 114.5 (quint., $^3J_{CF}$ = 4.6 Hz), 119.7, 133.9, 134.0, 141.4, 150.4, 152.8, 157.2 (quint., $^2J_{CF}$ = 17.2 Hz), 196.8; ^{19}F NMR (470.3 MHz, $CDCl_3$) δ_F 61.1 (d, 4F, $^2J_{FF}$ = 149.8 Hz), 83.1 (quint., 1F, $^2J_{FF}$ = 149.8 Hz); MS (EI) *m*/*z* (rel. int.) 414 (18), 413 (86) $[M]^+$, 412 (55), 398 (12), 383 (18), 382 (100), 370 (9), 338 (9), 286 (11, 246 (17), 195 (16), 138 (10); HRMS (ESI) *m*/*z* calcd for $C_{16}H_{17}F_5NO_4S$ $[M + H]^+$ 414.07930, found 414.07926.

4. Synthesis of ethyl 2-methyl-4-phenyl-6-(pentafluorosulfanyl)quinoline-3-carboxylate (12)

A mixture of **10a** (90 mg, 0.28 mmol), ethyl acetoacetate (109 mg, 0.84 mmol, 3 equiv), CAN (15 mg, 0.03 mmol, 0.1 equiv) and methanol (0.7 mL) was stirred at rt for 24 h. Water (40 mL) was added, and the crude product was extracted into EtOAc (3 × 15 mL). The combined organic phase was washed with brine (20 mL) and dried, and the solvent was removed under reduced pressure. Flash chromatography using silica gel (EtOAc–PE, 15:85) provided pure **12** as a colorless film (107 mg, 92% yield). R_f 0.45 (EtOAc–PE, 20:80); IR

(film) ν_{max} 3090, 3062, 3035, 1730, 1641, 1613, 1583, 1564, 1486, 1446, 1401, 1385, 1313, 1296, 1229, 1093, 1069, 959, 854, 815; ^{1}H NMR (500 MHz, $CDCl_3$) δ_H 0.96 (t, 3H, $^3J_{HH}$ = 7.1 Hz), 2.82 (s, 3H), 4.09 (q, 2H, $^3J_{HH}$ = 7.1 Hz), 7.36 (m, 2H), 7.53 (m, 3H), 8.02 (br d, 1H, $^4J_{HH}$ = 2.5 Hz), 8.04 (dd, 1H, $^3J_{HH}$ = 9.1 Hz, $^4J_{HH}$ = 2.5 Hz), 8.14 (br d, 1H, $^3J_{HH}$ = 9.1 Hz); ^{13}C NMR (125.7 MHz, $CDCl_3$) δ_C 13.6, 24.0, 61.6, 124.2, 125.2 (quint., $^3J_{CF}$ = 4.8 Hz), 126.7 (quint., $^3J_{CF}$ = 4.1 Hz), 128.6, 128.8, 129.2, 129.8, 134.3, 147.3, 147.8, 151.4 (quint., $^2J_{CF}$ = 17.3 Hz), 157.8, 167.7; ^{19}F NMR (376 MHz, $CDCl_3$) δ_F 62.9 (d, 4F, $^2J_{FF}$ = 150.2 Hz), 83.4 (quint., 1F, $^2J_{FF}$ = 150.2 Hz); MS (EI) *m*/*z* (rel. int.) 418 (14), 417 (65) $[M]^+$, 473 (21), 472 (100), 471 (39), 217 (35); HRMS (ESI) *m*/*z* calcd for $C_{19}H_{17}F_5NO_2S$ $[M + H]^+$ 418.08947, found 418.08892.

5. Synthesis of 9-phenyl-6-(pentafluorosulfanyl)-1,2,3,4-tetrahydroacridine (13)

A mixture of **11a** (148 mg, 0.46 mmol), cyclohexanone (270 mg, 2.75 mmol, 6 equiv), CAN (50 mg, 0.09 mmol, 0.2 equiv) and methanol (1.8 mL) was stirred at rt for 48 h. Water (60 mL) was added, and the crude product was extracted into EtOAc (3 × 30 mL). The combined organic phase was washed with brine (30 mL) and dried, and the solvent was removed under reduced pressure. Flash chromatography using silica gel (EtOAc–PE, 5:95) provided pure **13** as a colorless film (110 mg, 62% yield). R_f 0.32 (EtOAc–PE, 5:95); IR (film) ν_{max} 3060, 3030, 2941, 2866, 1579, 1561, 1499, 1485, 1434, 1358, 1351, 1082, 937, 898, 849, 815; ^{1}H NMR (500 MHz, $CDCl_3$) δ_H 1.81 (m, 2H), 1.98 (m, 2H), 2.65 (t, 2H, $^3J_{HH}$ = 6.5 Hz), 3.22 (t, 2H, $^3J_{HH}$ = 6.6 Hz), 7.22 (m, 2H), 7.41 (br d, 1H, $^3J_{HH}$ = 9.2 Hz), 7.49 (m, 1H), 7.54 (m, 2H), 7.62 (dd, 1H, $^3J_{HH}$ = 9.2 Hz, $^4J_{HH}$ = 2.3 Hz), 8.50 (d, 1H, $^4J_{HH}$ = 2.3 Hz); ^{13}C NMR (125.7 MHz, $CDCl_3$) δ_C 22.6, 22.7, 28.1, 34.2, 121.7 (quint., $^3J_{CF}$ = 4.2 Hz), 126.5, 127.1 (quint., $^3J_{CF}$ = 4.7 Hz), 127.7, 128.2, 128.8, 128.9, 131.2, 136.0, 145.0, 146.2, 153.0 (quint., $^2J_{CF}$ = 17.3 Hz), 161.5; ^{19}F NMR (376 MHz, $CDCl_3$) δ_F 62.8 (d, 4F, $^2J_{FF}$ = 150.1 Hz), 83.7 (quint., 1F, $^2J_{FF}$ = 150.1 Hz); MS (EI) *m*/*z* (rel. int.) 386 (21), 385 (100) $[M]^+$, 384 (34), 258 (39); HRMS (CI) *m*/*z* calcd for $C_{19}H_{17}F_5NS$ $[M + H]^+$ 386.1002, found 386.1008.

6. Synthesis of 2,4-diphenyl-7-(pentafluorosulfanyl)quinazoline (14)

A mixture of **11a** (90 mg, 0.28 mmol), benzylamine (210 mg, 2 mmol, 7 equiv), I_2 (7 mg, 0.03 mmol, 0.1 equiv) and *t*-BuOOH (220 mg, 2 mmol, 7 equiv, 80% aq solution) was heated at 90 °C for 24 h. Water (40 mL) was added, and the crude product was extracted into EtOAc (3 × 20 mL). The combined organic phase was washed with brine (30 mL) and dried, and the

solvent was removed under reduced pressure. Flash chromatography using silica gel (EtOAc–PE, 5:95) provided pure **14** as a white solid (76 mg, 66% yield). mp 166–167 °C; R_f 0.56 (EtOAc–PE, 5:95); IR (film) ν_{max} 3092, 3062, 3040, 2928, 1612, 1601, 1580, 1565, 1538, 1483, 1347, 1082, 929, 851, 817; ^{1}H NMR (500 MHz, $CDCl_3$) δ_H 7.52 (m, 3H), 7.61 (m, 3H), 7.83 (dd, 1H, $^3J_{HH}$ = 9.1 Hz, $^4J_{HH}$ = 2.3 Hz), 7.86 (m, 2H), 8.19 (d, 1H, $^3J_{HH}$ = 9.1 Hz), 8.57 (dd, 1H, $^4J_{HH}$ = 2.3 Hz, $^5J_{HH}$ = 0.5 Hz), 8.68 (m, 2H); ^{13}C NMR (125.7 MHz, $CDCl_3$) δ_C 122.3, 123.3 (quint., $^3J_{CF}$ = 4.2 Hz), 127.8 (quint., $^3J_{CF}$ = 4.6 Hz), 128.0, 128.6, 128.8, 128.8, 130.1, 130.5, 131.2, 136.7, 137.2, 151.5, 156.9 (quint., $^2J_{CF}$ = 18.1 Hz), 161.6, 168.4; ^{19}F NMR (376 MHz, $CDCl_3$) δ_F 62.0 (d, 4F, $^2J_{FF}$ = 150.3 Hz), 81.8 (quint., 1F, $^2J_{FF}$ = 150.3 Hz); MS (EI) *m*/*z* (rel. int.) 409 (19), 408 (80) $[M]^+$, 407 (54), 282 (24), 281 (100), 178 (13), 177 (12), 151 (13); HRMS (ESI) *m*/*z* calcd for $C_{20}H_{14}F_5N_2S$ $[M + H]^+$ 409.07924, found 409.07927.

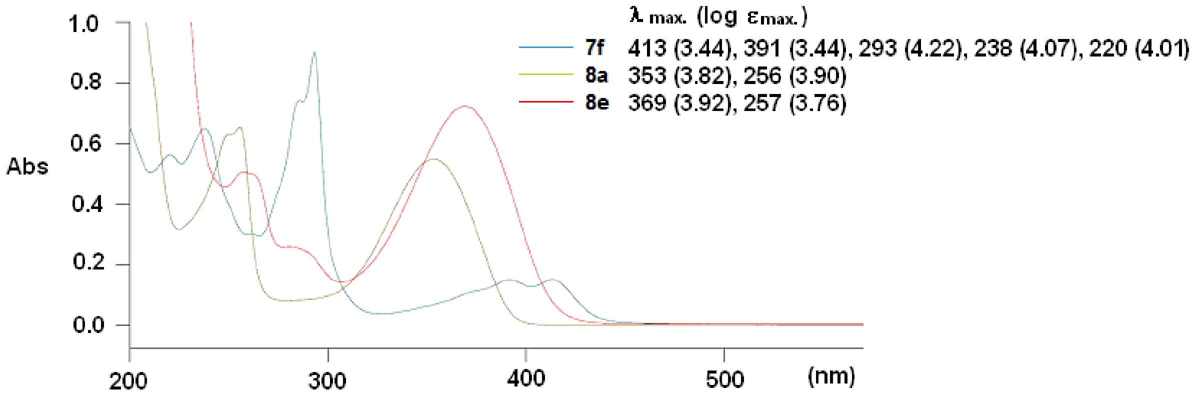

Figure S1: UV–vis absorption spectra of benzisoxazoles **7f**, **8a**, and **8e** in $CHCl_3$.

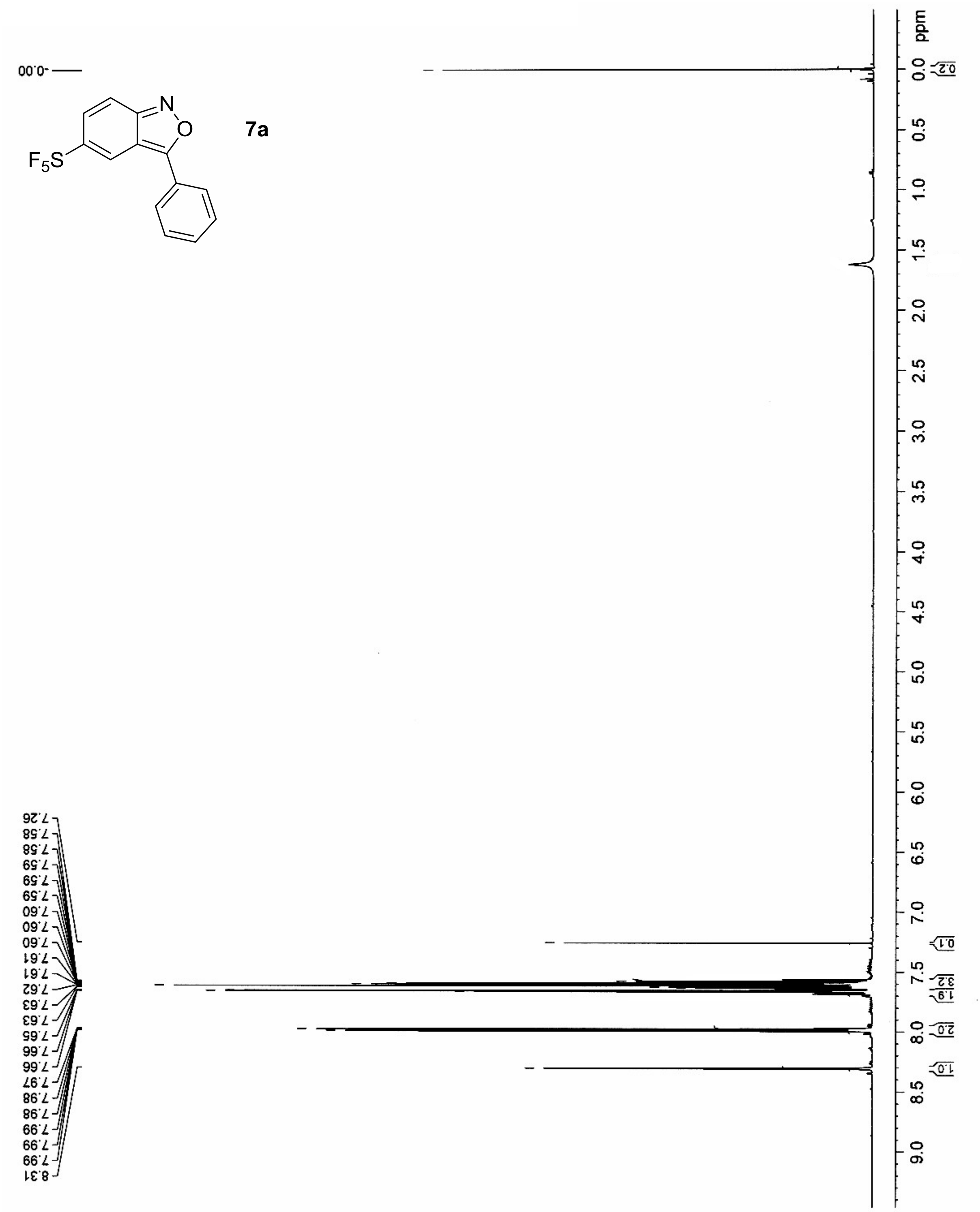
7a
ppm

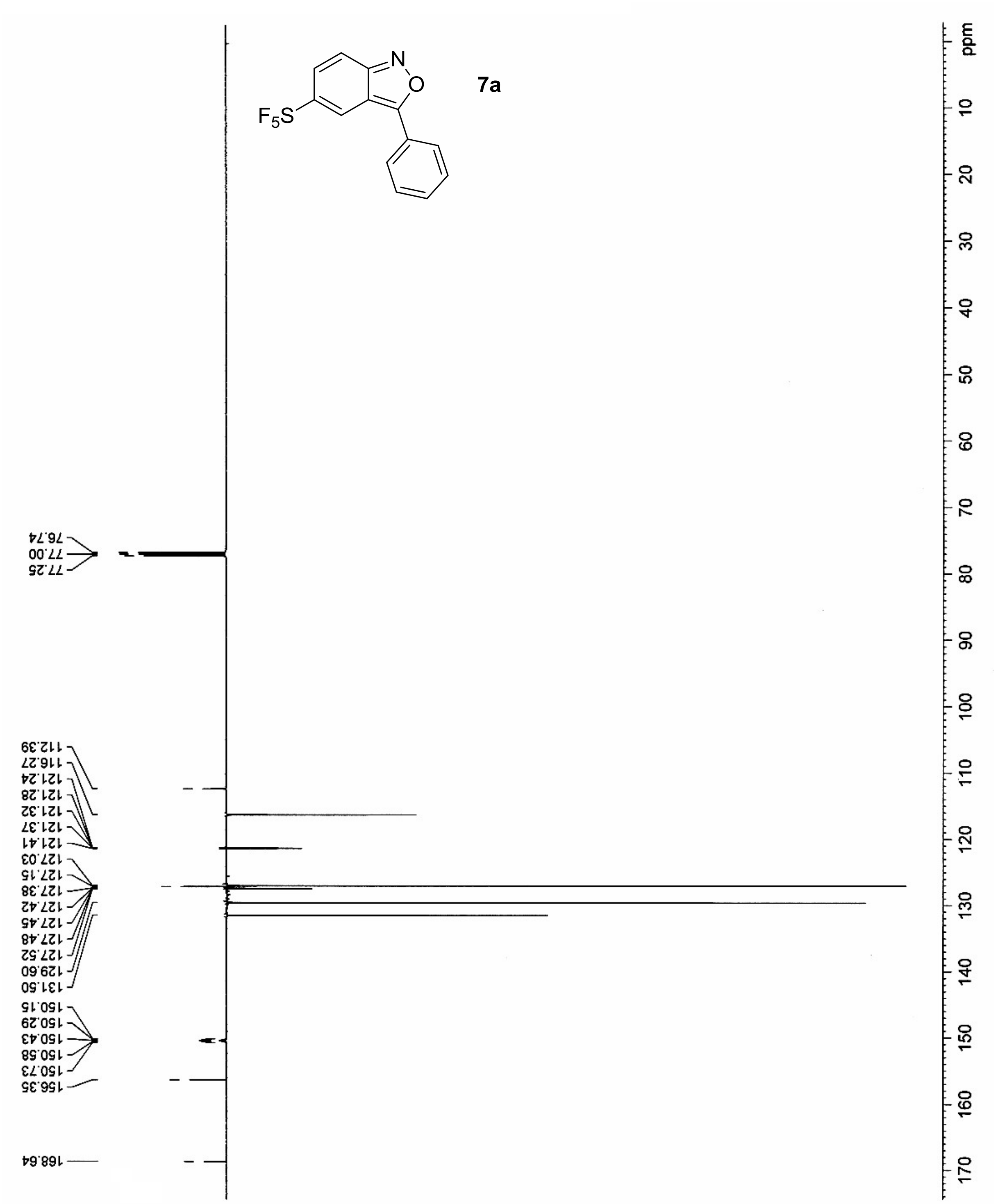

F5S
N
O
7a
ppm
76.74
77.00
77.25
112.39
116.27
121.24
121.28
121.32
121.37
121.41
127.03
127.15
127.38
127.42
127.45
127.48
127.52
129.60
131.50
150.15
150.29
150.43
150.58
150.73
156.35
168.64

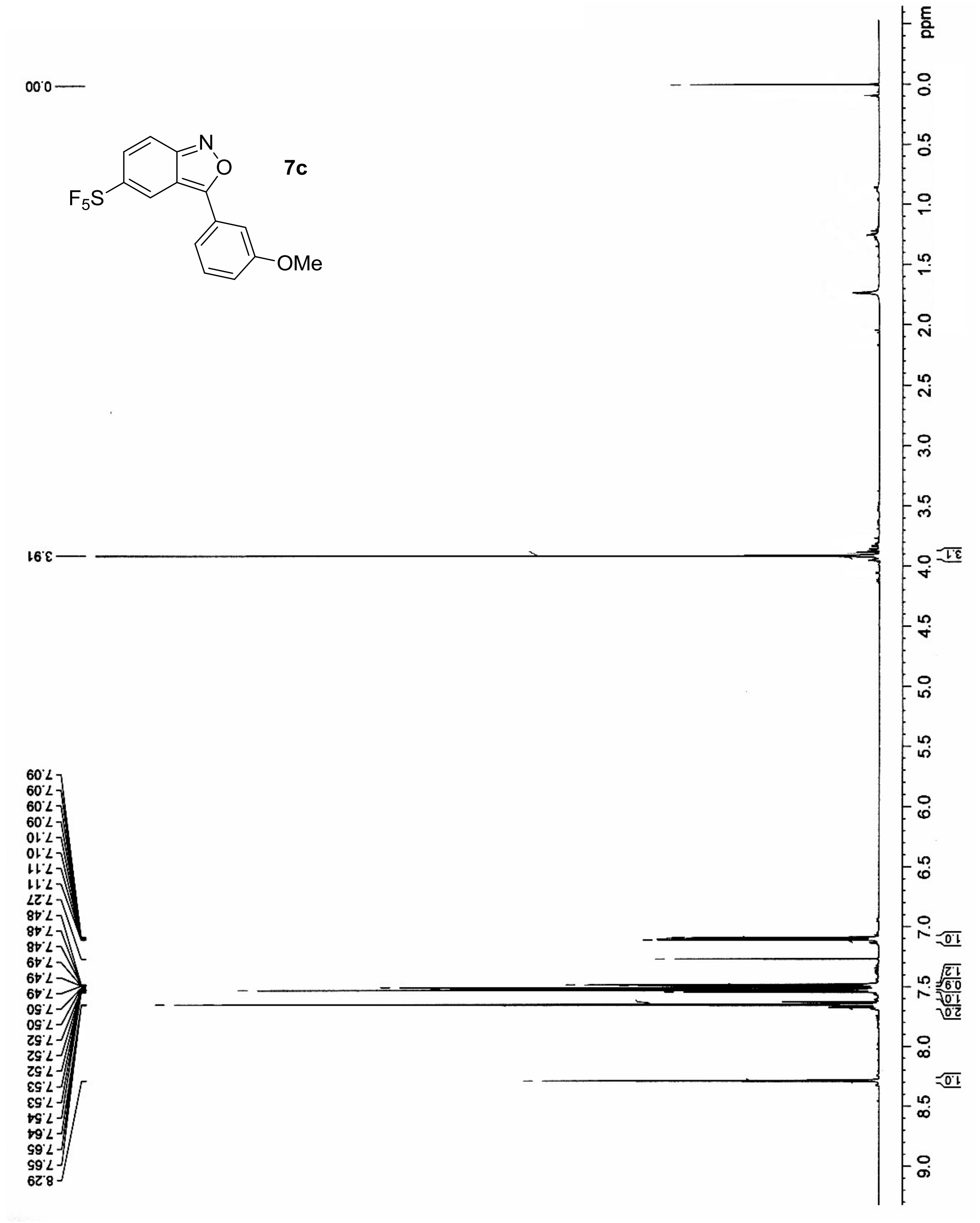
0.00
3.91
7.09
7.09
7.09
7.09
7.10
7.10
7.11
7.11
7.27
7.48
7.48
7.48
7.49
7.49
7.49
7.50
7.50
7.52
7.52
7.52
7.53
7.53
7.54
7.64
7.65
7.65
8.29
N
O
F_5S
OMe
7c
ppm
3.1
1.0
1.2
0.9
1.0
2.0
1.0

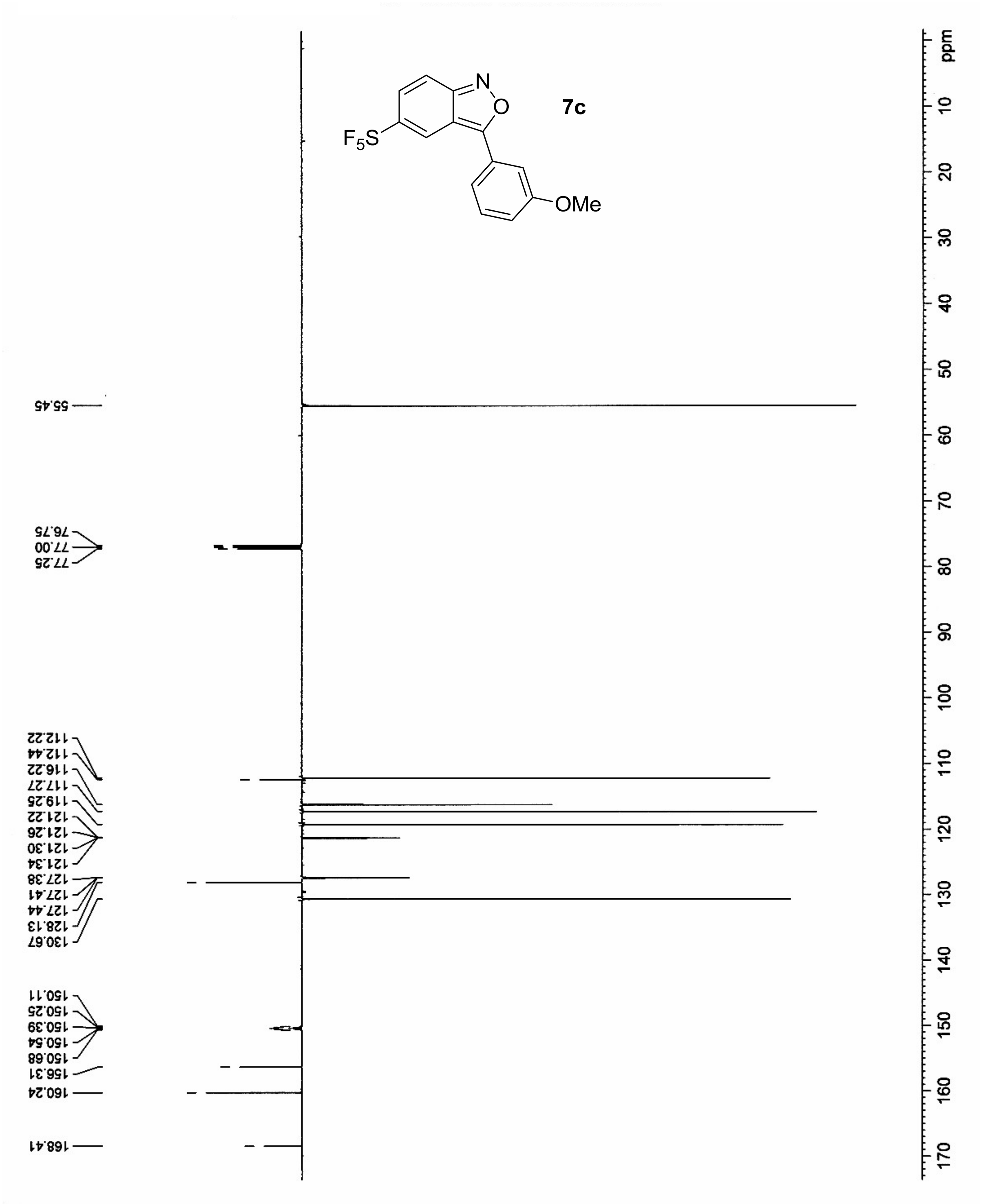

7c
N
O
F5S
OMe
ppm
55.45
76.75
77.00
77.25
112.22
112.44
116.22
117.27
119.25
121.22
121.26
121.30
121.34
127.38
127.41
127.44
128.13
130.67
150.11
150.25
150.39
150.54
150.68
156.31
160.24
168.41

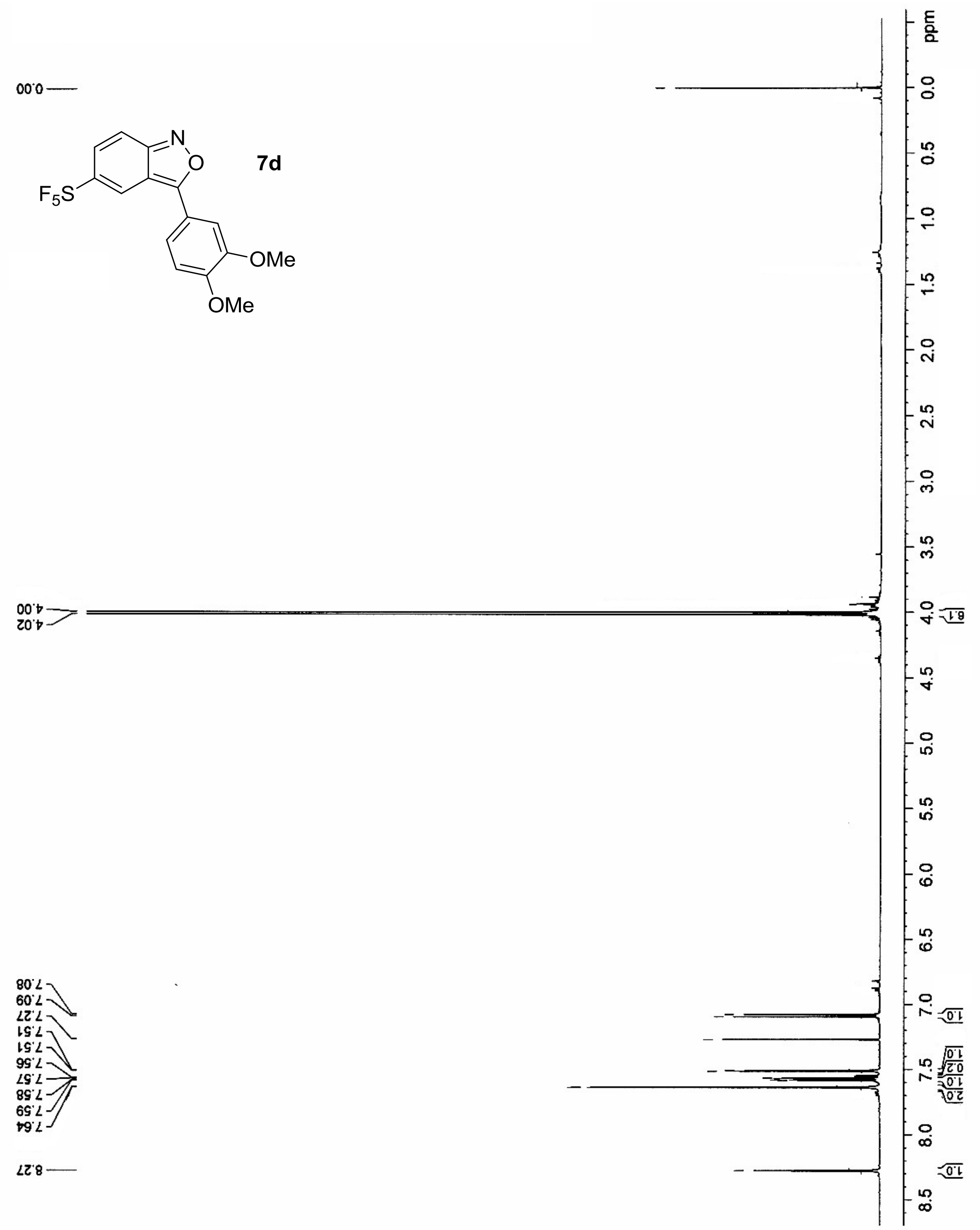
7d
F5S
OMe
OMe
0.00
4.00
4.02
7.08
7.09
7.27
7.51
7.51
7.56
7.57
7.58
7.59
7.64
8.27
ppm

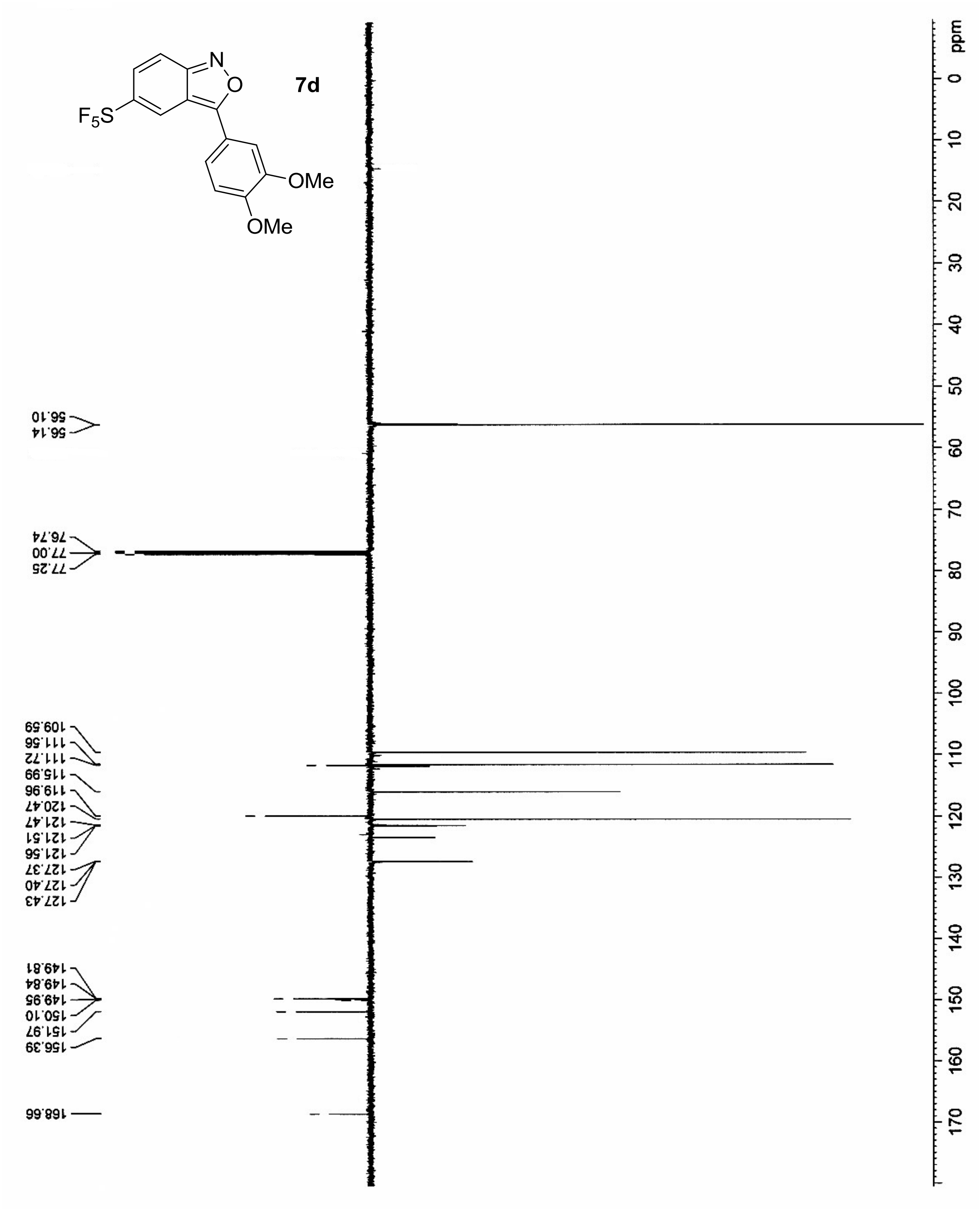
F5S
N
O
OMe
OMe
7d
ppm
56.10
56.14
76.74
77.00
77.25
109.59
111.56
111.72
115.99
119.96
120.47
121.47
121.51
121.56
127.37
127.40
127.43
149.81
149.84
149.95
150.10
151.97
156.39
168.66

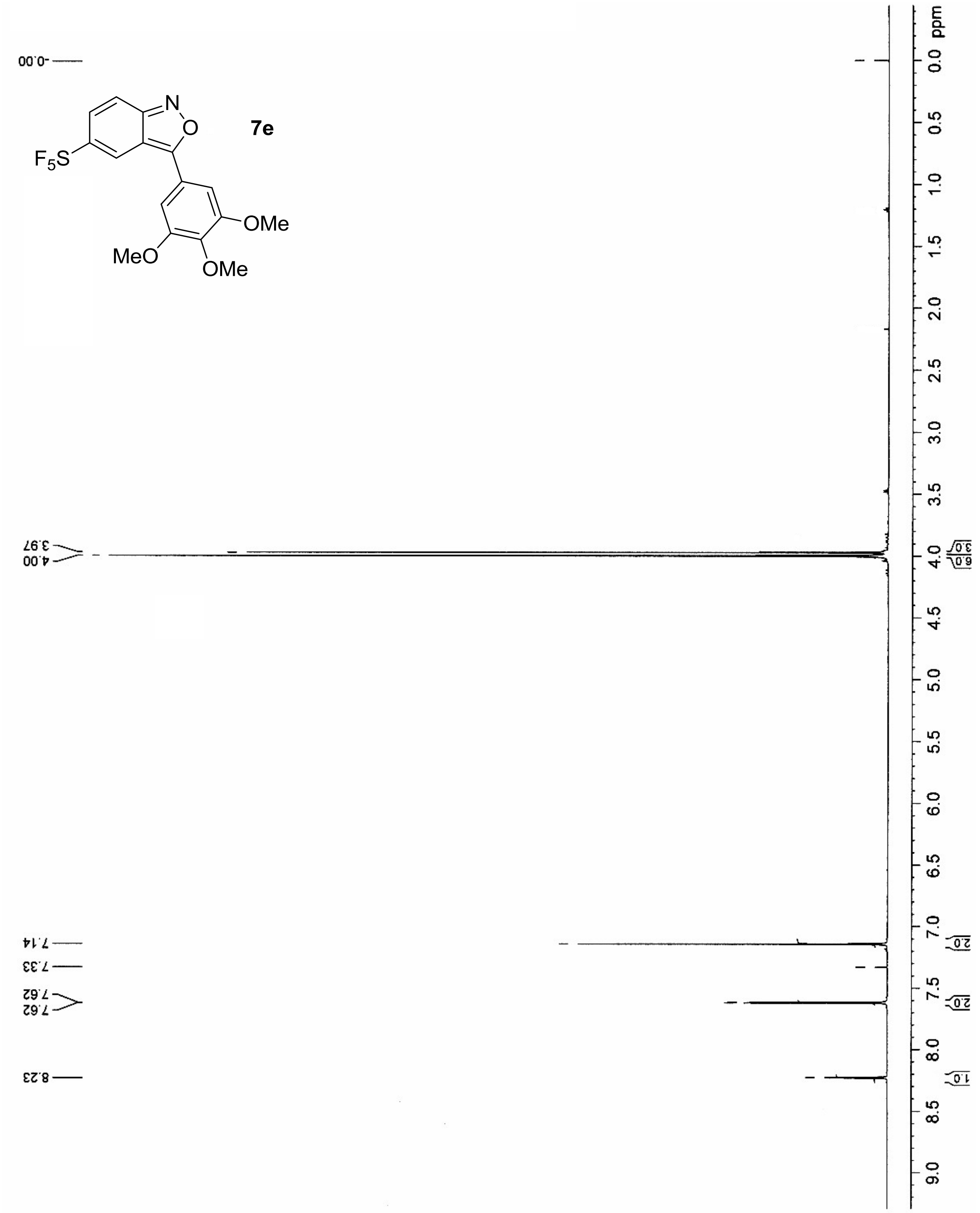
7e
F5S
N
O
OMe
MeO
OMe
-0.00
3.97
4.00
7.14
7.33
7.62
7.62
8.23
ppm
3.0
6.0
2.0
2.0
1.0

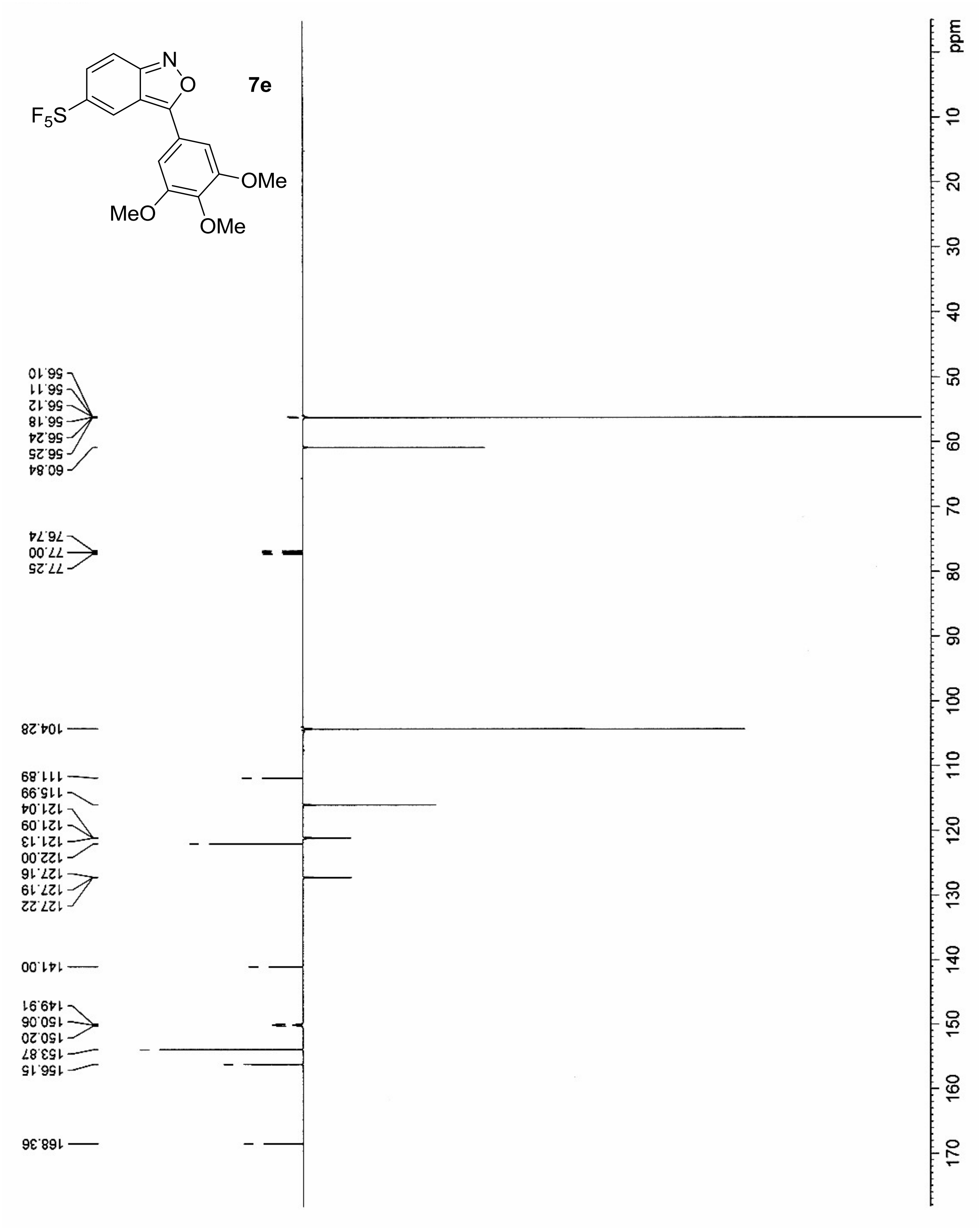
7e
F5S
N
O
OMe
MeO
OMe

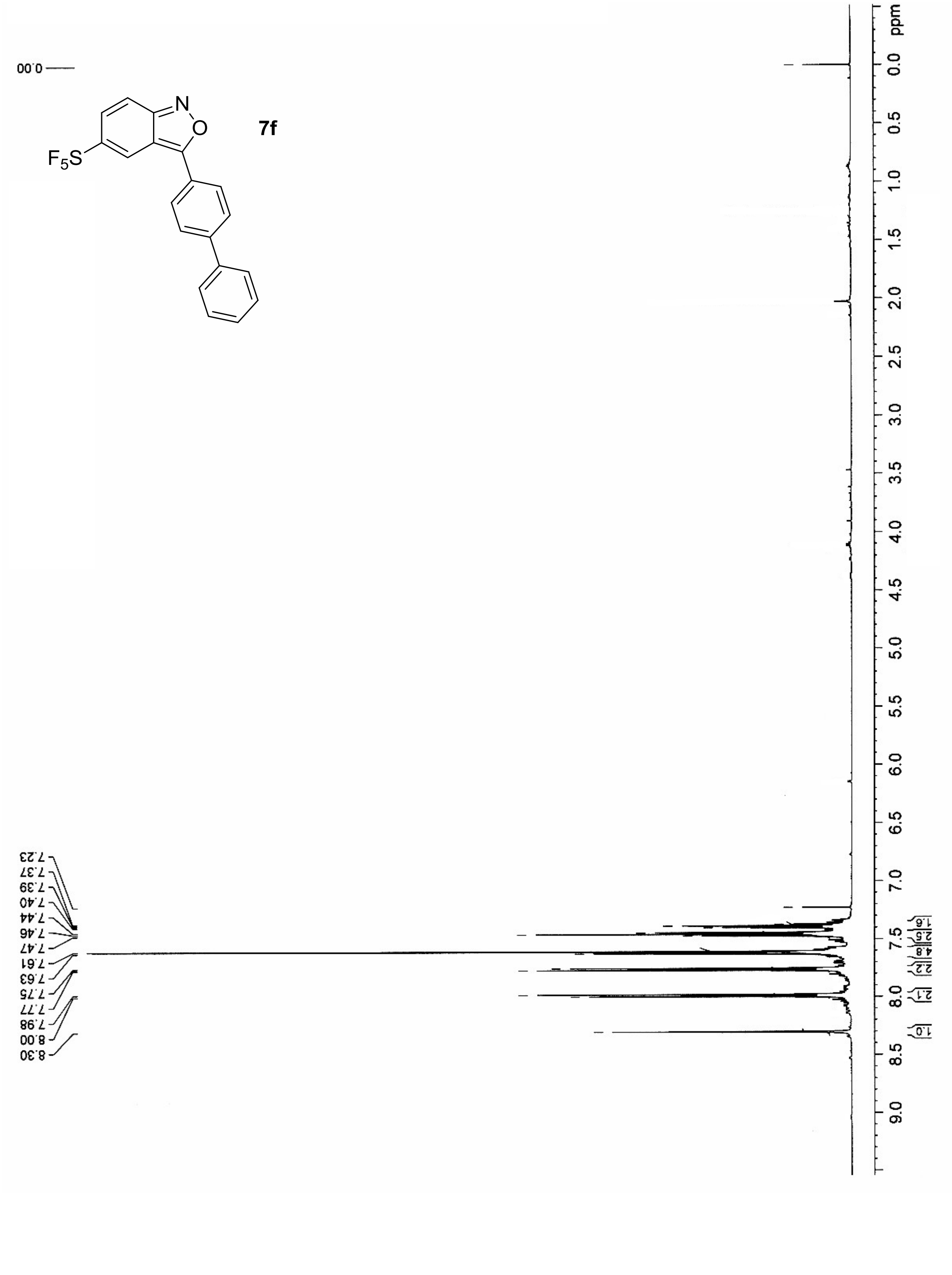
7f
F5S
N
O
0.00
7.23
7.37
7.39
7.40
7.44
7.46
7.47
7.61
7.63
7.75
7.77
7.98
8.00
8.30
1.0
2.1
2.2
4.8
2.5
1.6
ppm

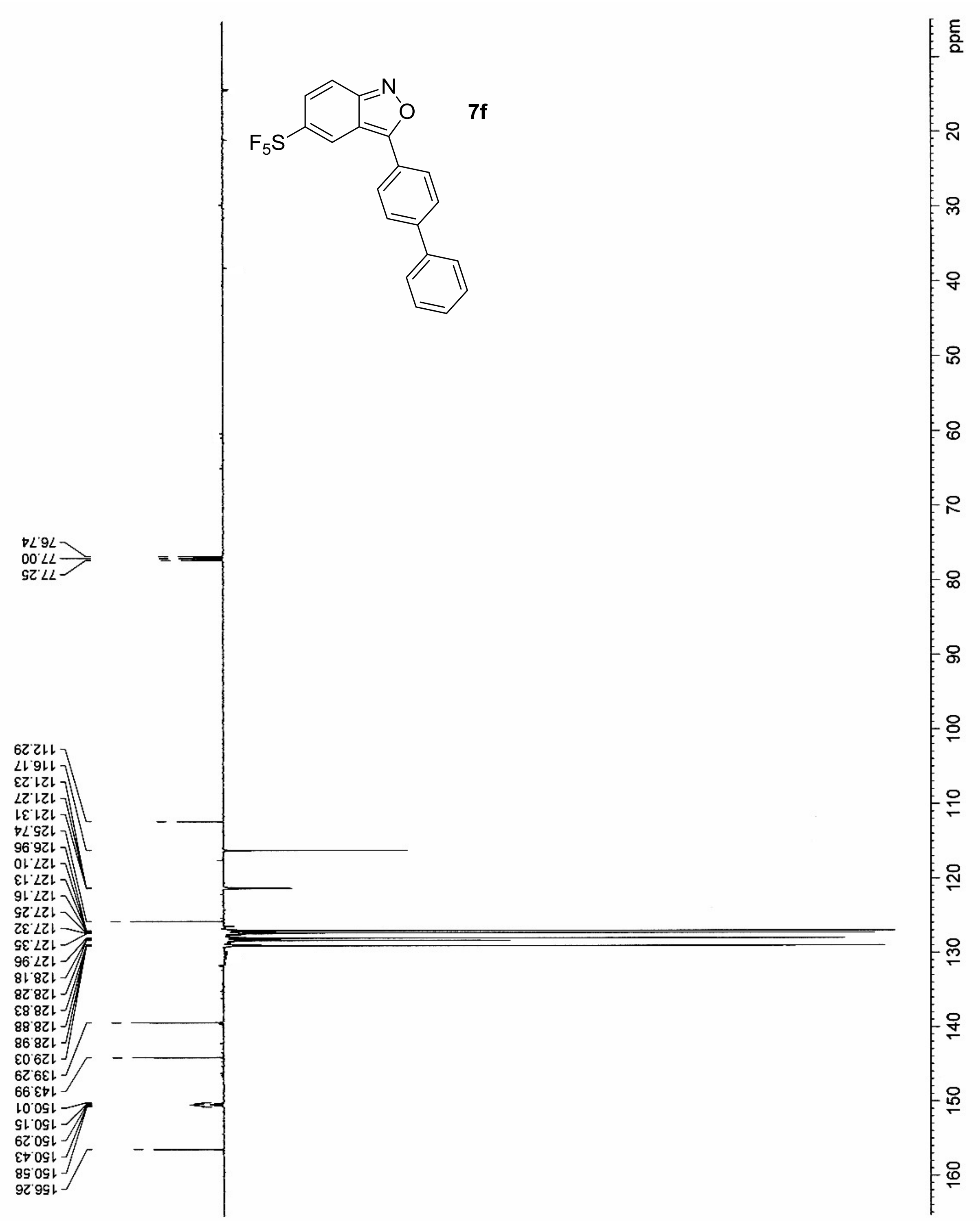
7f
F5S
N
O
ppm
76.74
77.00
77.25
112.29
116.17
121.23
121.27
121.31
125.74
126.96
127.10
127.13
127.16
127.25
127.32
127.35
127.96
128.18
128.28
128.83
128.88
128.98
129.03
139.29
143.99
150.01
150.15
150.29
150.43
150.58
156.26

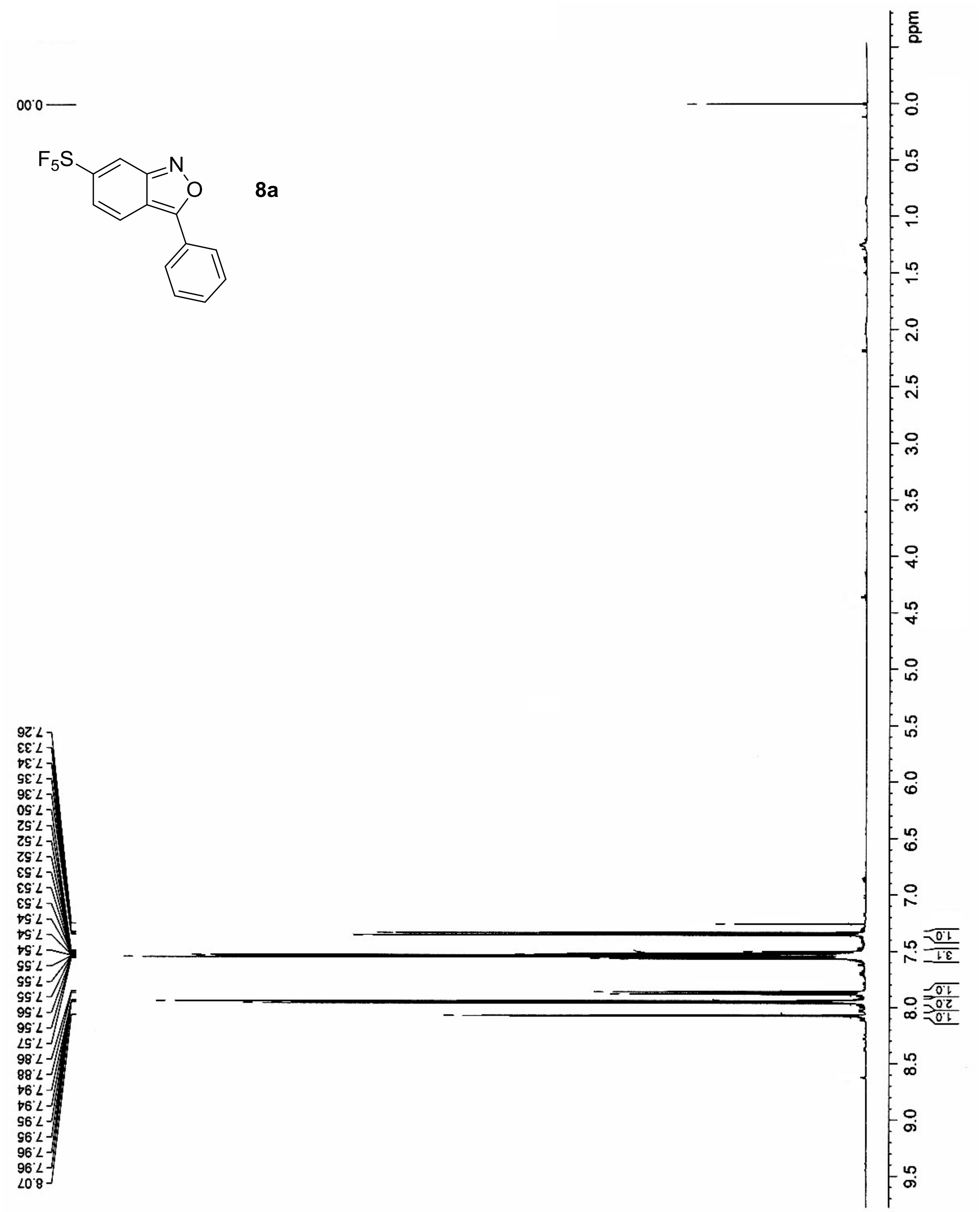
8a
ppm

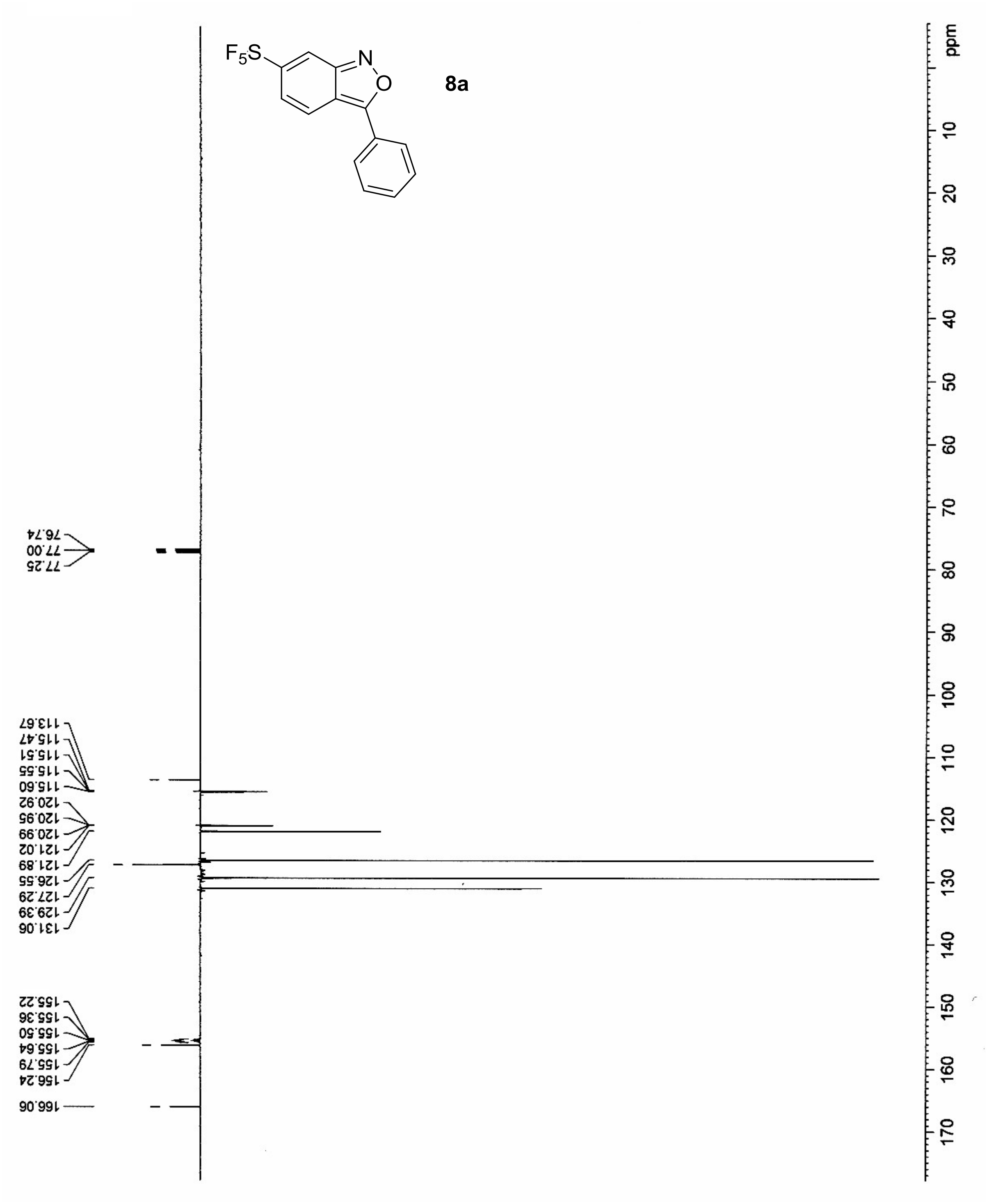
F5S
N
O
8a
ppm
76.74
77.00
77.25
113.67
115.47
115.51
115.55
115.60
120.92
120.95
120.99
121.02
121.89
126.55
127.29
129.39
131.06
155.22
155.36
155.50
155.64
155.79
156.24
166.06

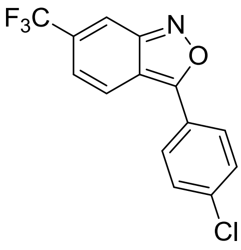

9b

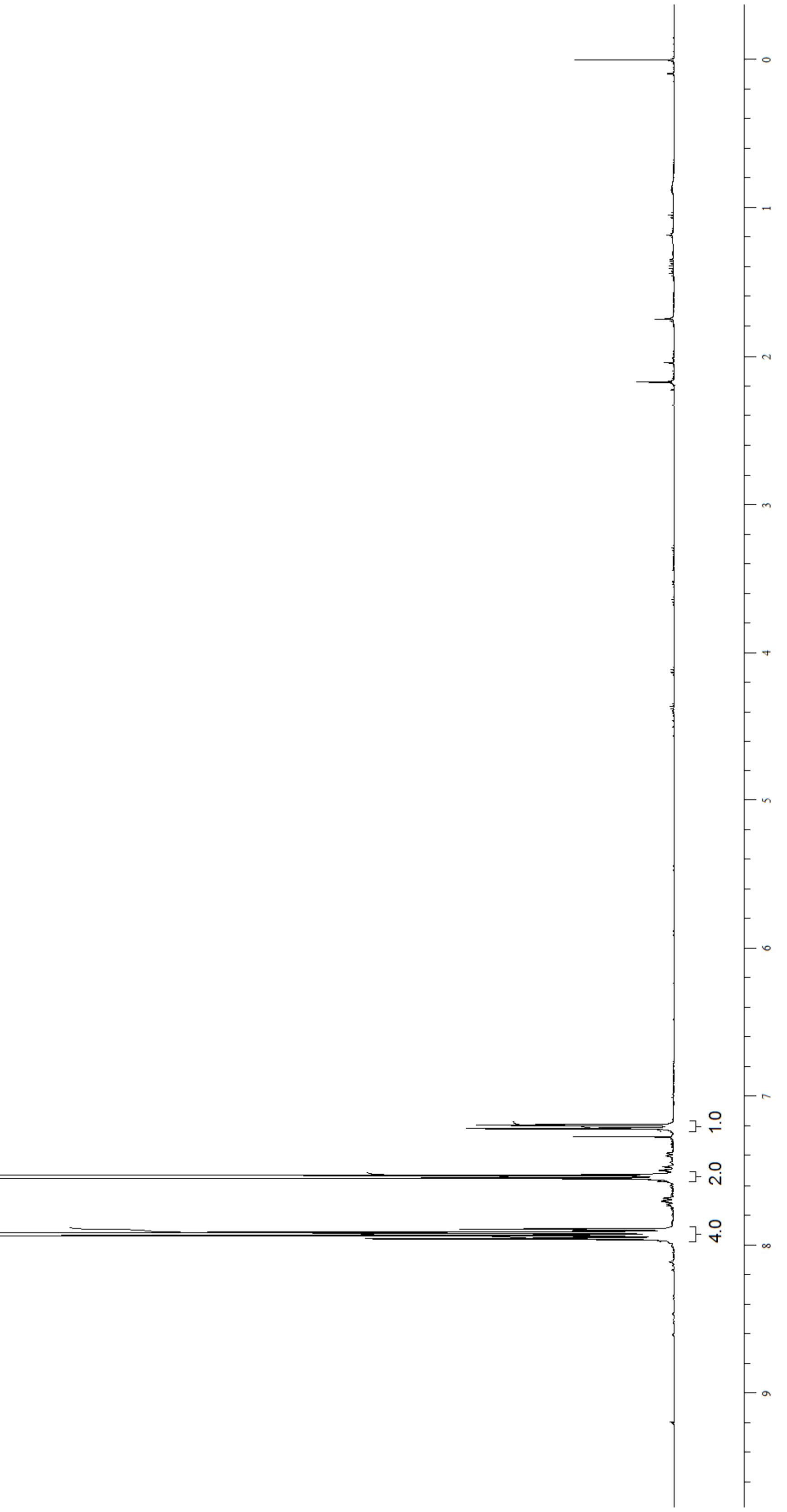

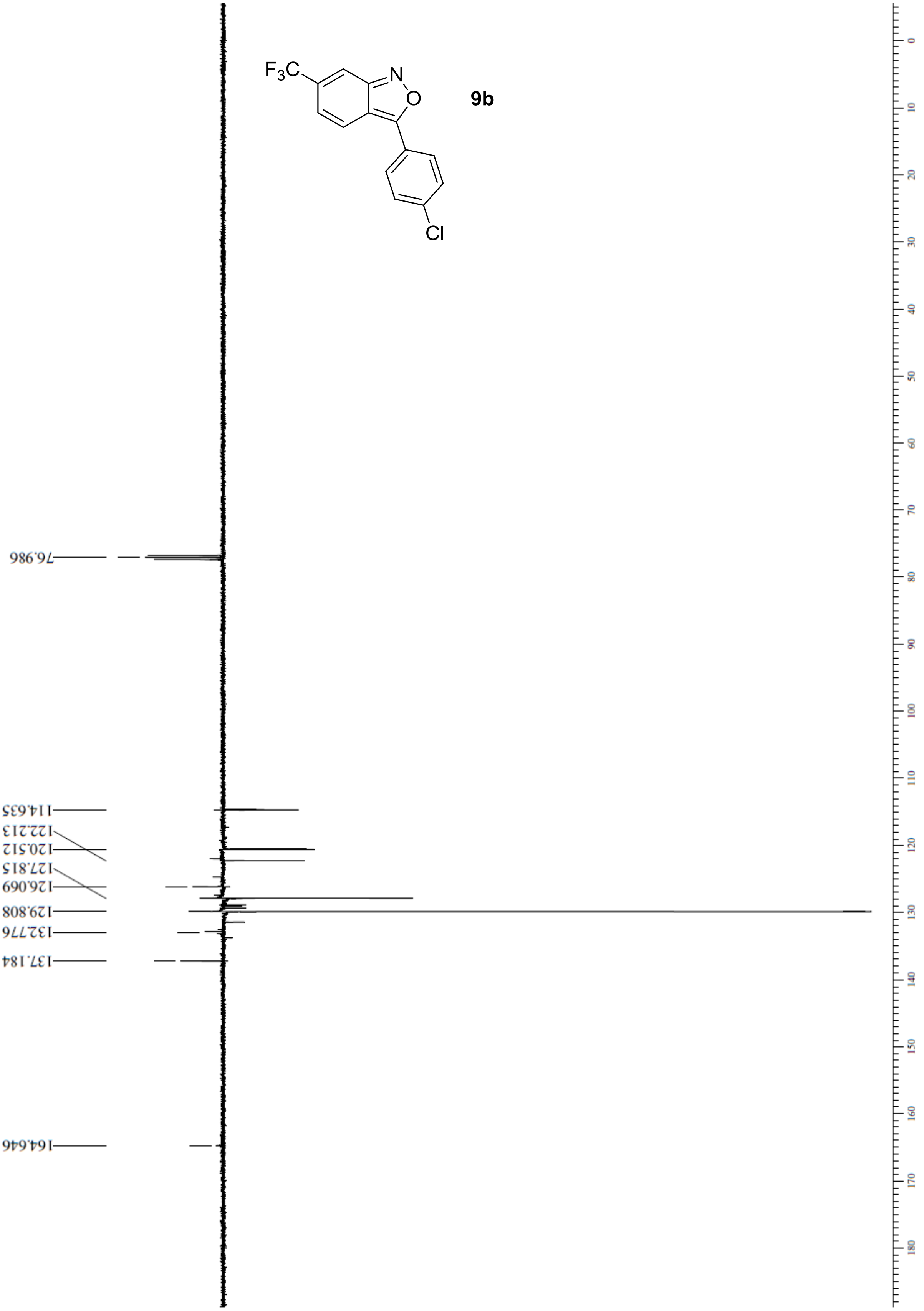
F3C
N
O
Cl
9b
76.986
114.635
122.213
120.512
127.815
126.069
129.808
132.776
137.184
164.646
0
10
20
30
40
50
60
70
80
90
100
110
120
130
140
150
160
170
180

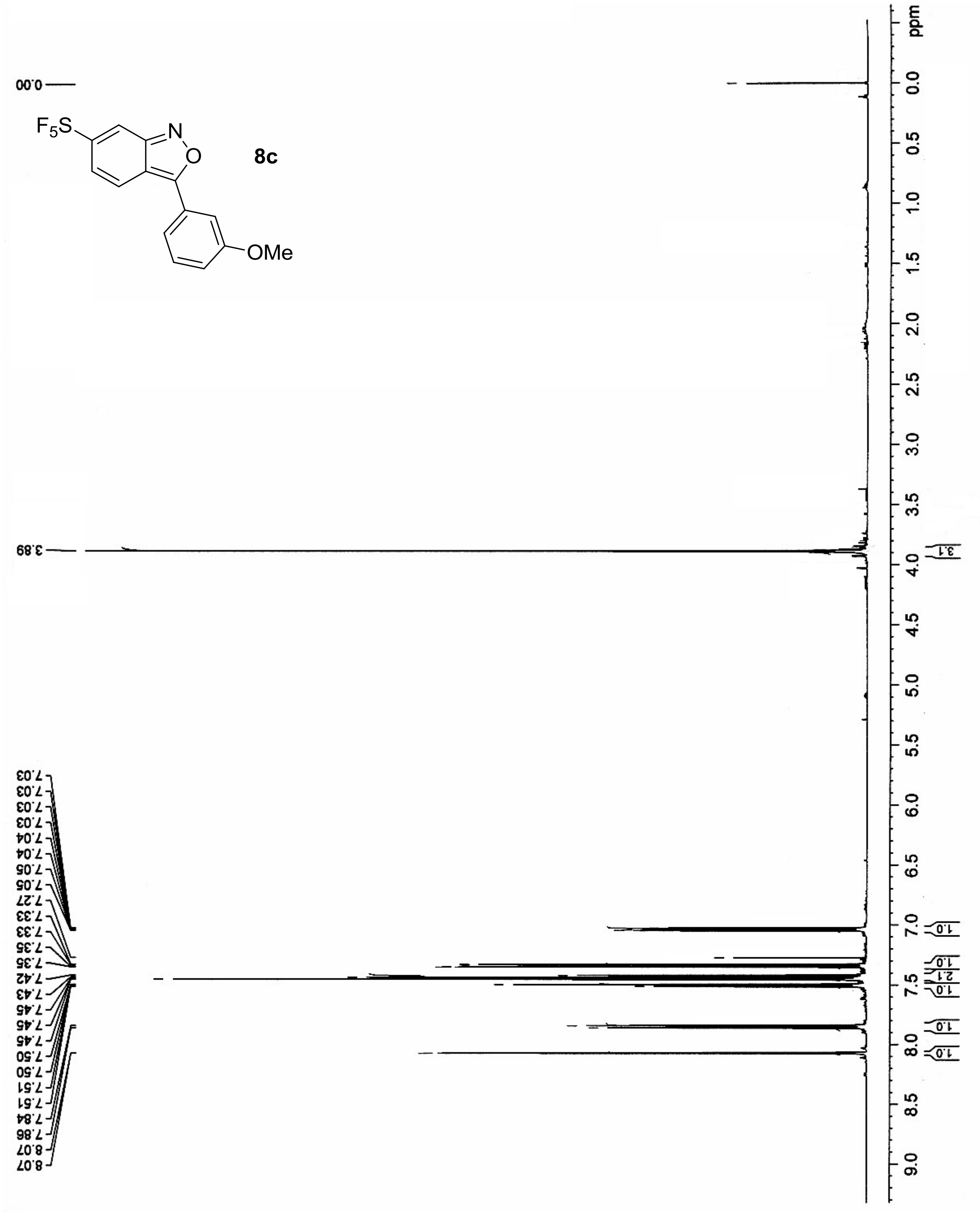
F5S
N
O
8c
OMe
0.00
3.89
7.03
7.03
7.03
7.03
7.04
7.04
7.05
7.05
7.27
7.33
7.33
7.35
7.35
7.42
7.43
7.45
7.45
7.45
7.50
7.50
7.51
7.51
7.84
7.86
8.07
8.07
ppm

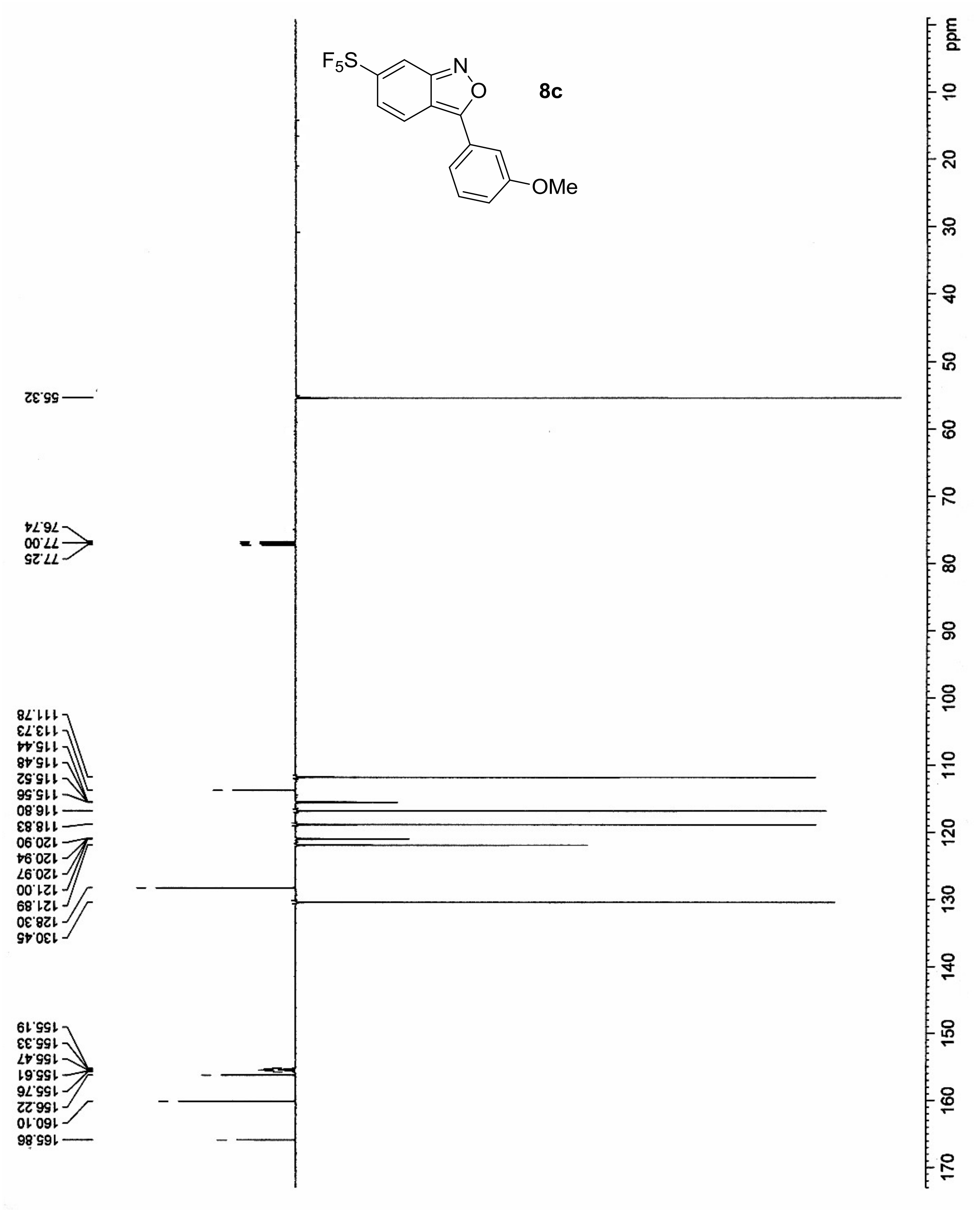

F5S
N
O
OMe
8c
ppm
10
20
30
40
50
60
70
80
90
100
110
120
130
140
150
160
170
55.32
76.74
77.00
77.25
111.78
113.73
115.44
115.48
115.52
115.56
116.80
118.83
120.90
120.94
120.97
121.00
121.89
128.30
130.45
155.19
155.33
155.47
155.61
155.76
156.22
160.10
165.86

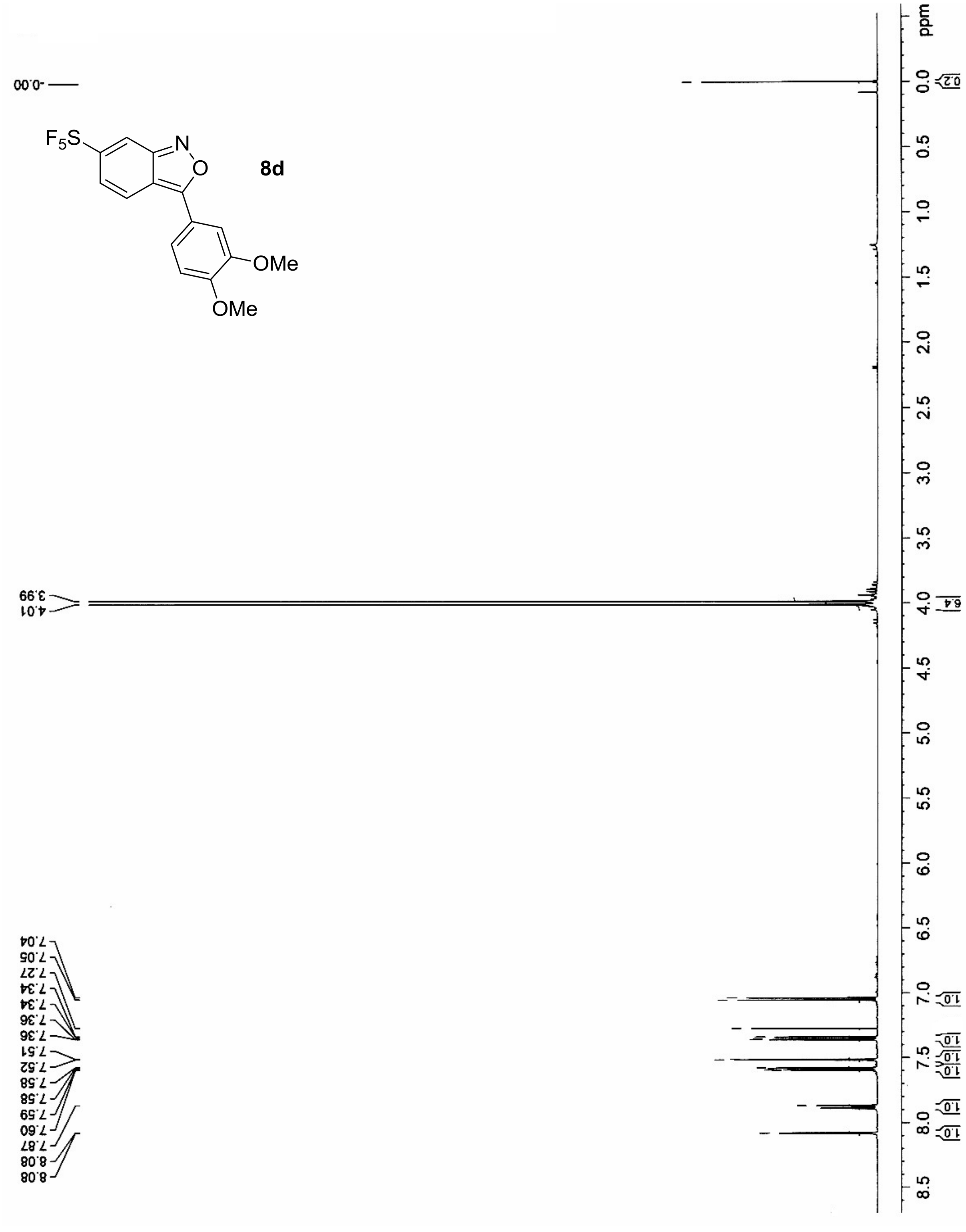
8d
ppm

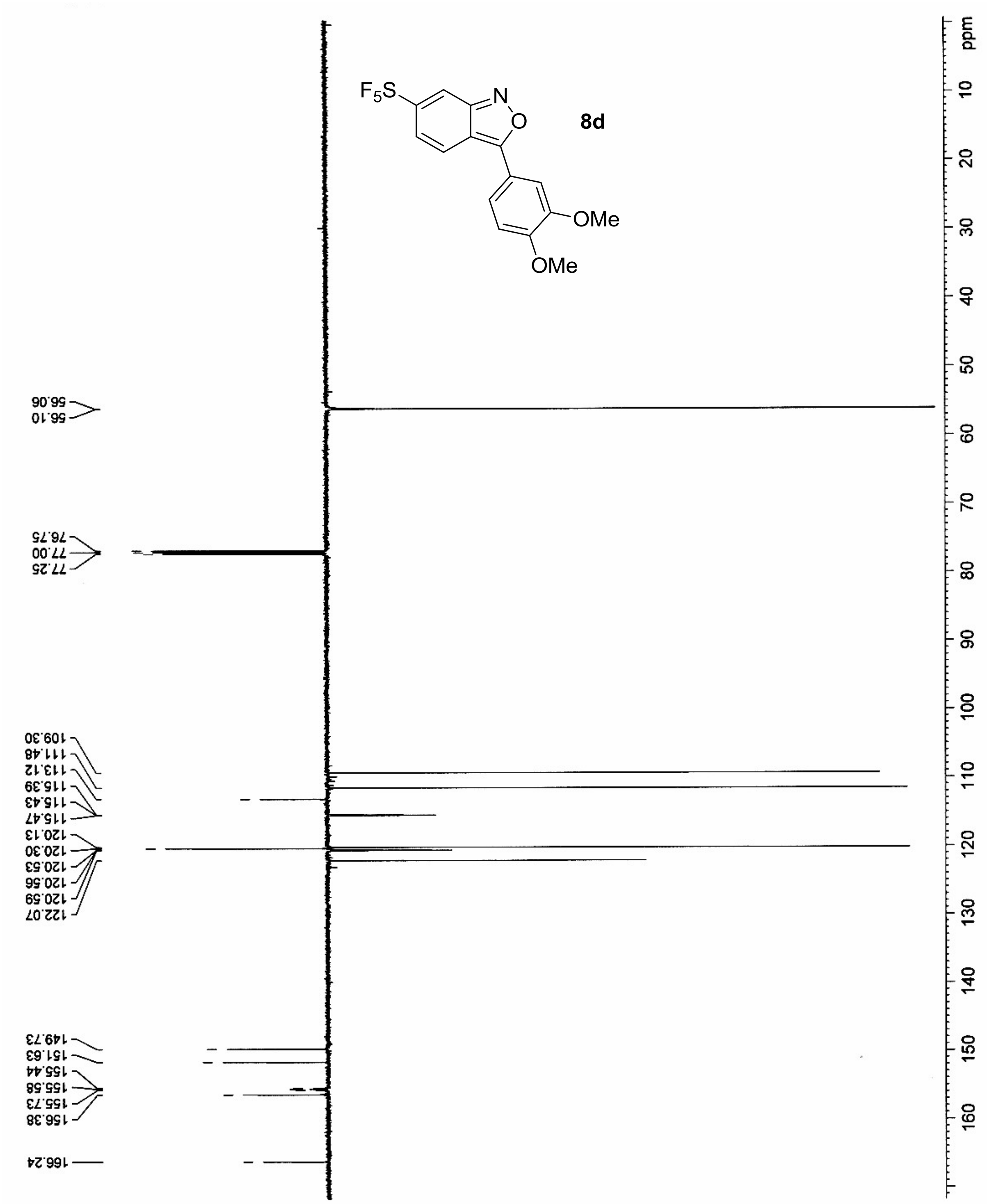
8d
F5S
N
O
OMe
OMe
ppm
56.06
56.10
76.75
77.00
77.25
109.30
111.48
113.12
115.39
115.43
115.47
120.13
120.30
120.53
120.56
120.59
122.07
149.73
151.63
155.44
155.58
155.73
156.38
166.24

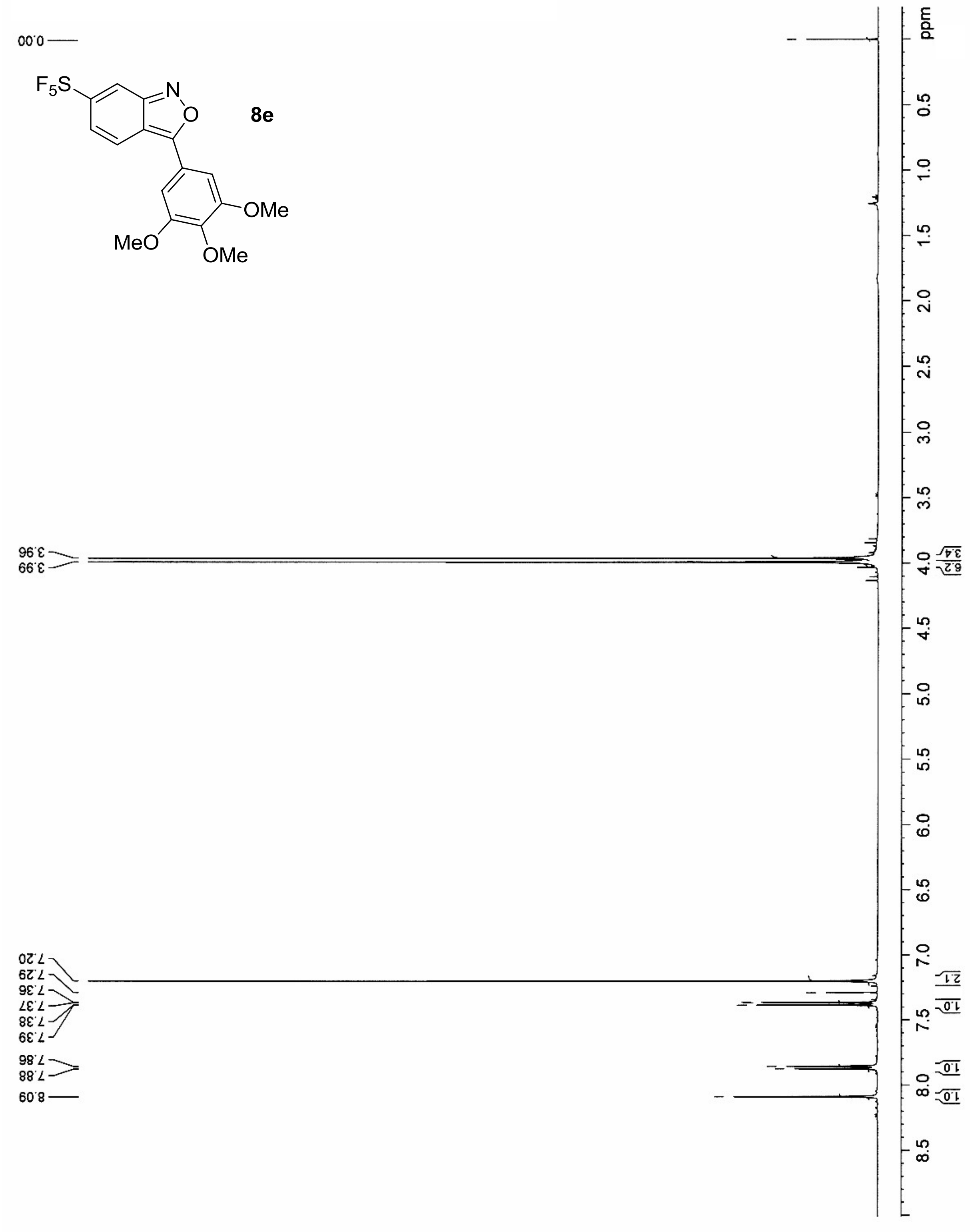

8e
F5S
N
O
OMe
MeO
OMe
0.00
3.96
3.99
7.20
7.29
7.36
7.37
7.38
7.39
7.86
7.88
8.09
ppm

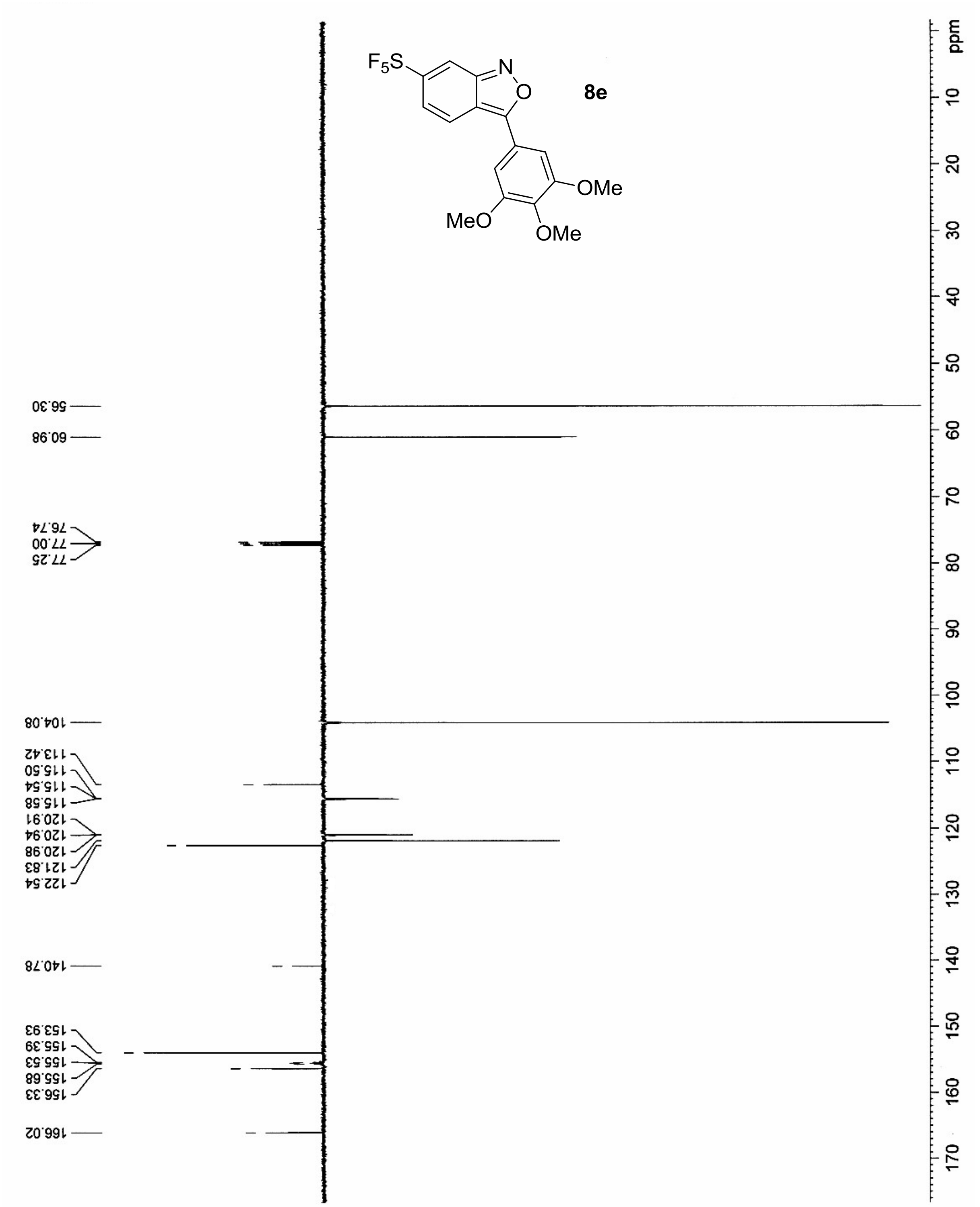
F5S
N
O
8e
OMe
MeO
OMe
ppm
56.30
60.98
76.74
77.00
77.25
104.08
113.42
115.50
115.54
115.58
120.91
120.94
120.98
121.83
122.54
140.78
153.93
155.39
155.53
155.68
156.33
166.02

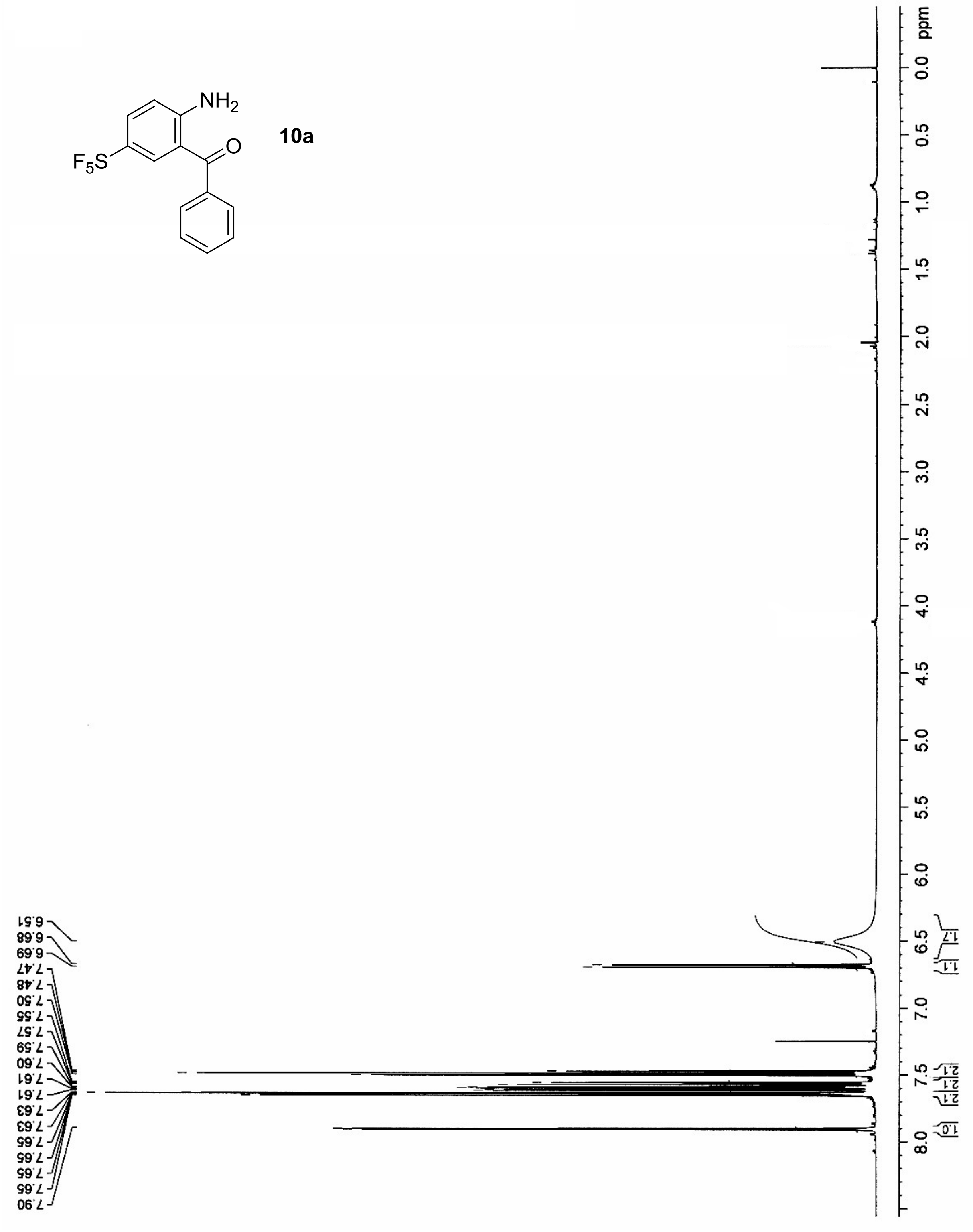

10a

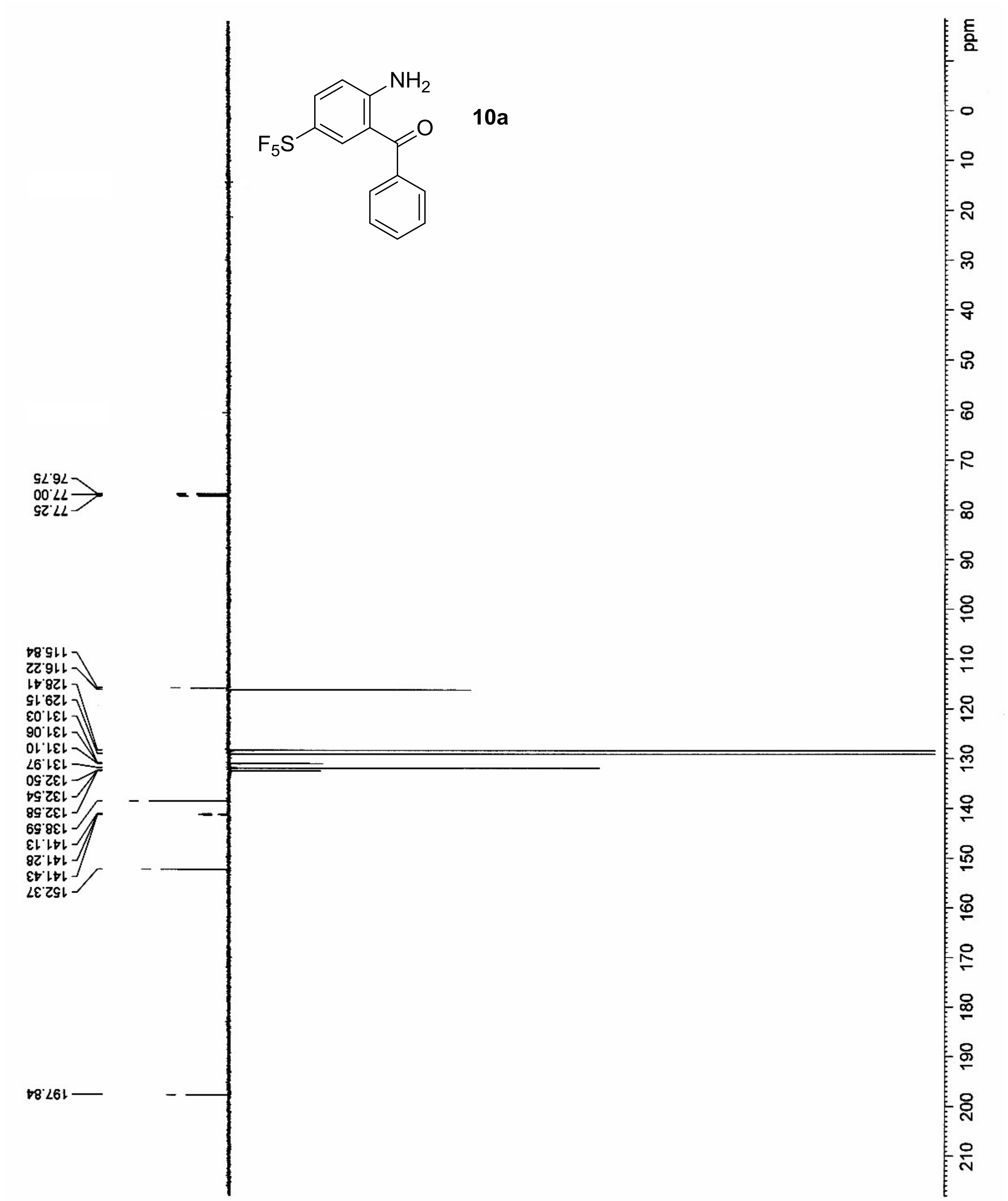
NH2
F5S
O
10a
ppm
76.75
77.00
77.25
115.84
116.22
128.41
129.15
131.03
131.06
131.10
131.97
132.50
132.54
132.58
138.59
141.13
141.28
141.43
152.37
197.84

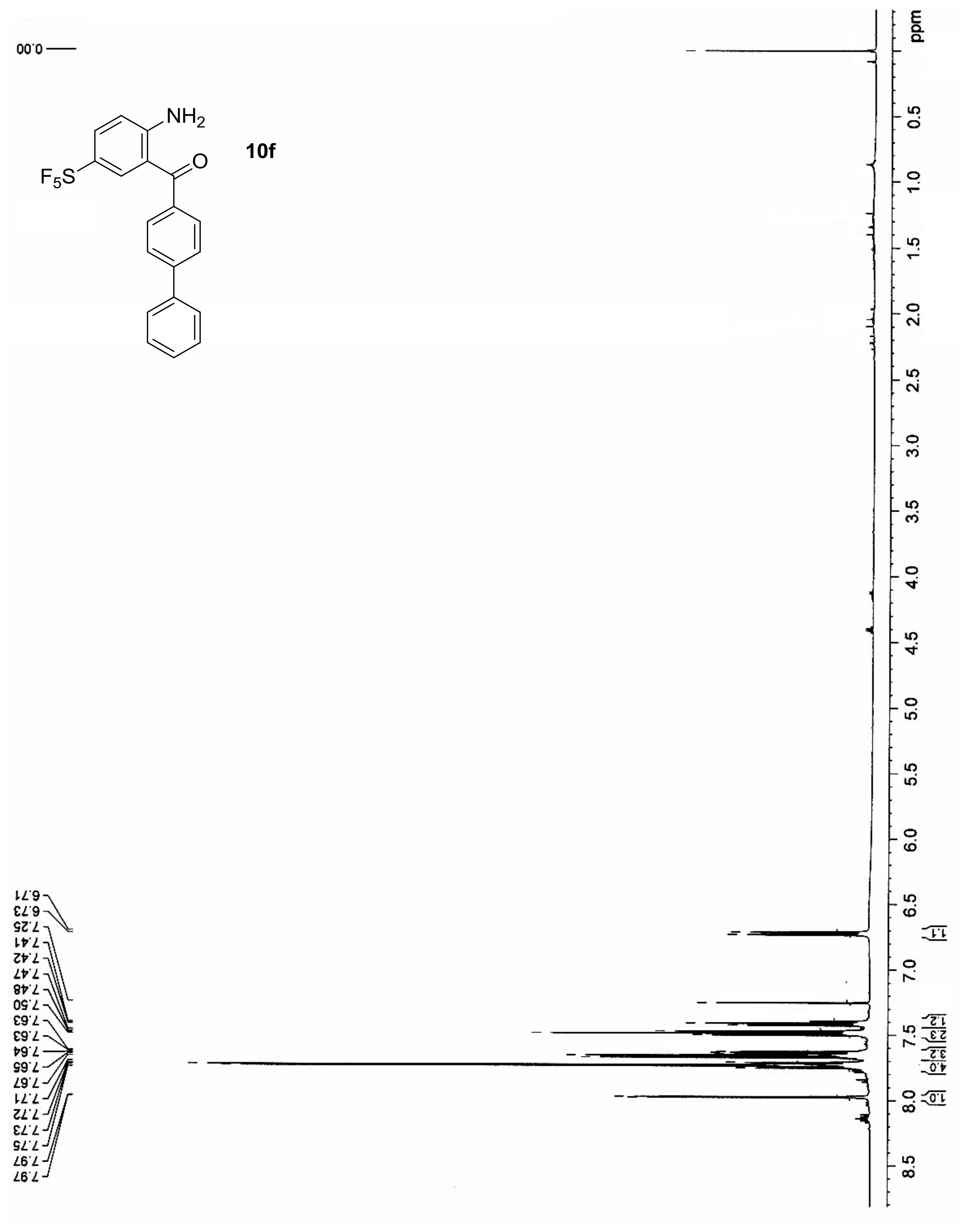
10f
ppm

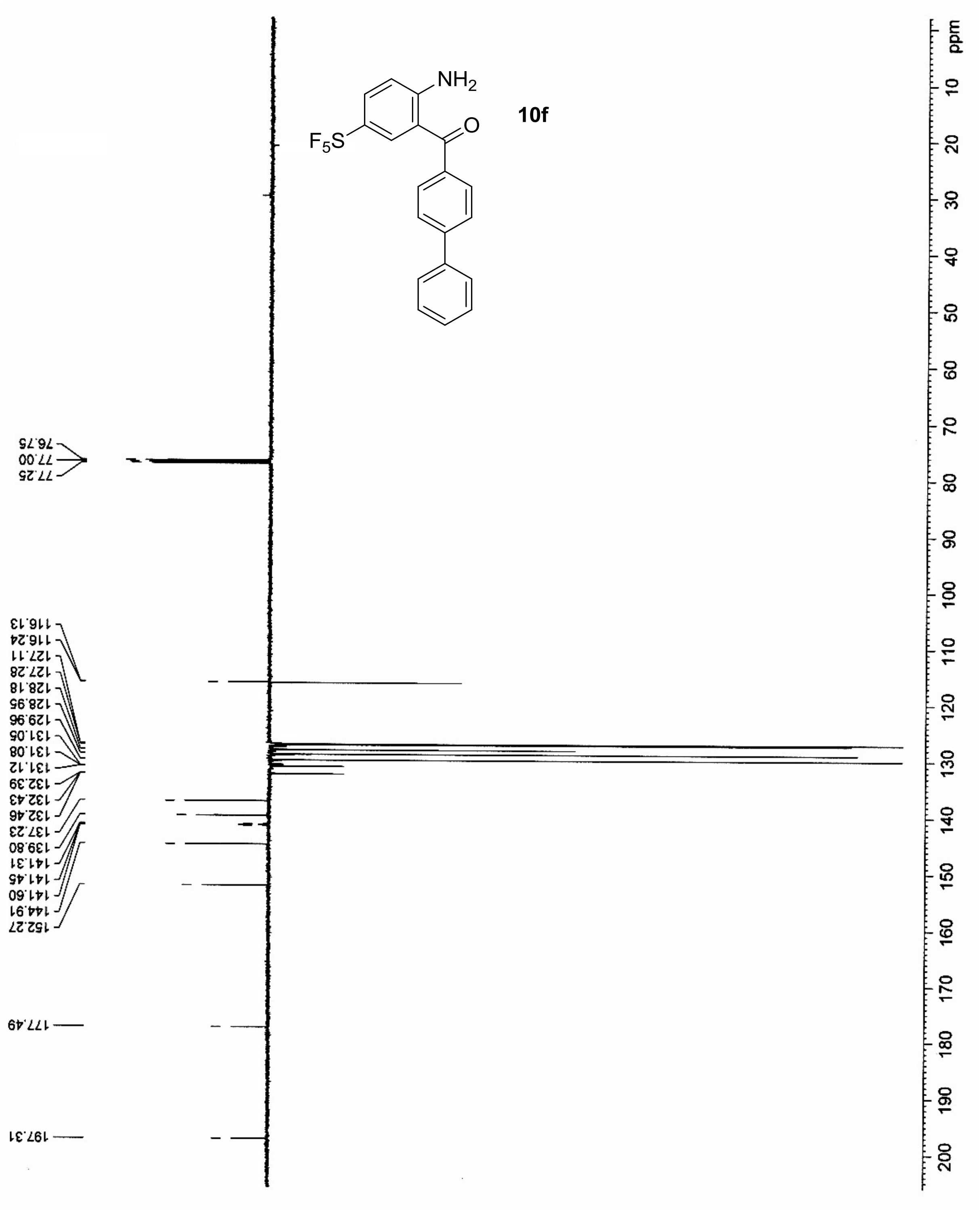
NH2
F5S
O
10f
76.75
77.00
77.25
116.13
116.24
127.11
127.28
128.18
128.95
129.96
131.05
131.08
131.12
132.39
132.43
132.46
137.23
139.80
141.31
141.45
141.60
144.91
152.27
177.49
197.31
ppm
10
20
30
40
50
60
70
80
90
100
110
120
130
140
150
160
170
180
190
200

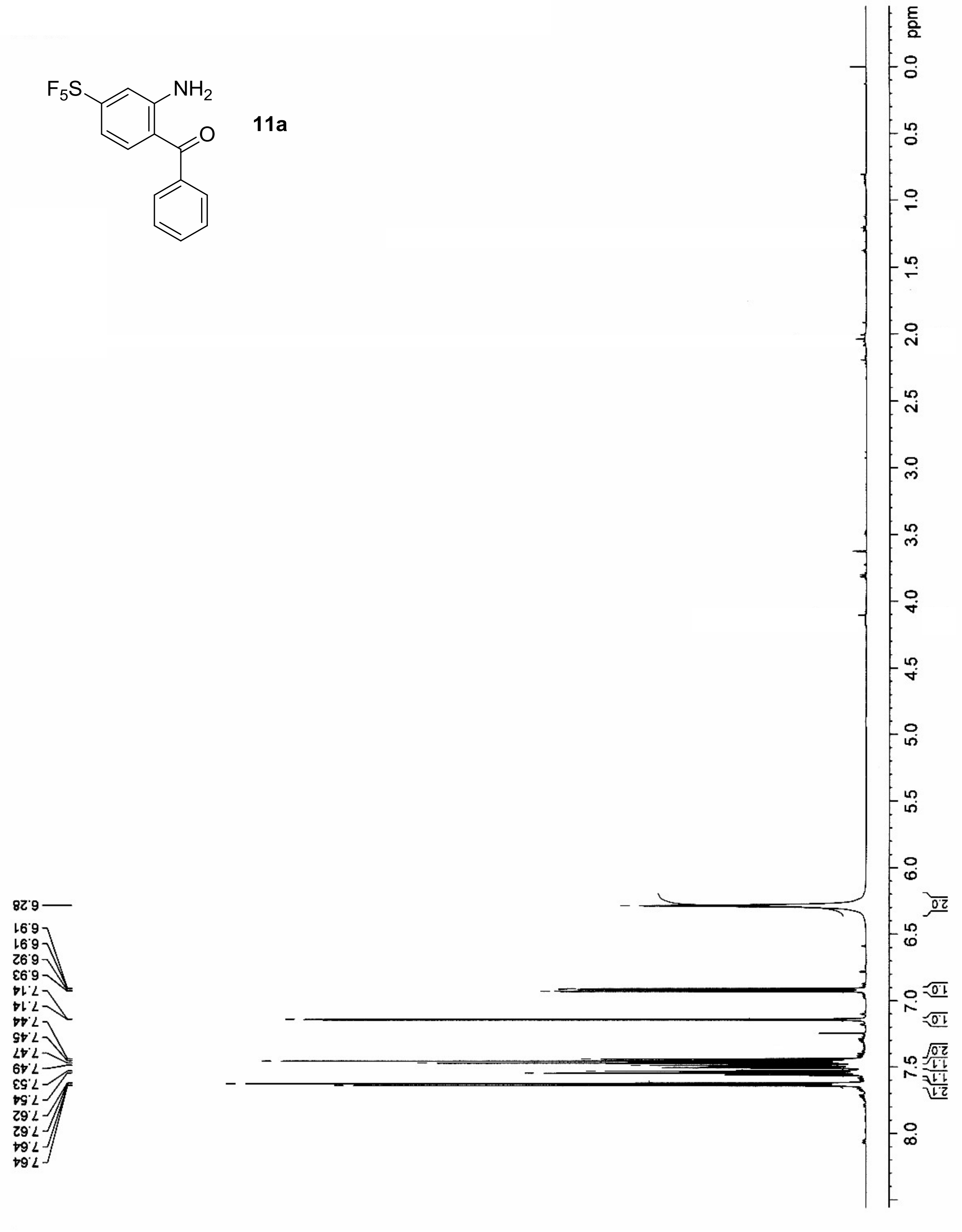
F5S
NH2
O
11a
6.28
6.91
6.91
6.92
6.93
7.14
7.14
7.44
7.45
7.47
7.49
7.53
7.54
7.62
7.62
7.64
7.64
2.0
1.0
1.0
2.0
1.1
1.1
2.1
ppm
0.0
0.5
1.0
1.5
2.0
2.5
3.0
3.5
4.0
4.5
5.0
5.5
6.0
6.5
7.0
7.5
8.0

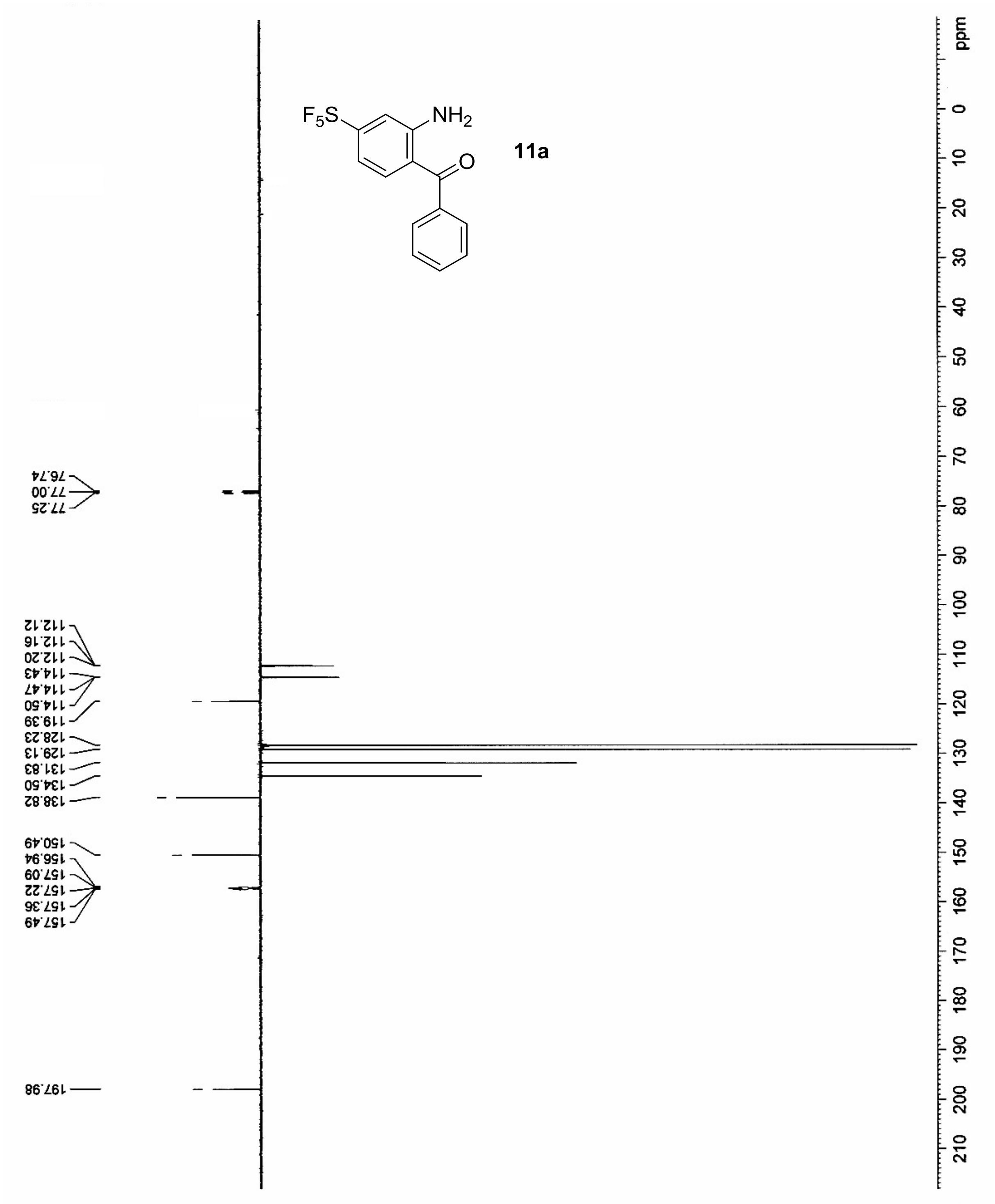
F5S
NH2
O
11a
ppm
0 10 20 30 40 50 60 70 80 90 100 110 120 130 140 150 160 170 180 190 200 210
76.74
77.00
77.25
112.12
112.16
112.20
114.43
114.47
114.50
119.39
128.23
129.13
131.83
134.50
138.82
150.49
156.94
157.09
157.22
157.36
157.49
197.98

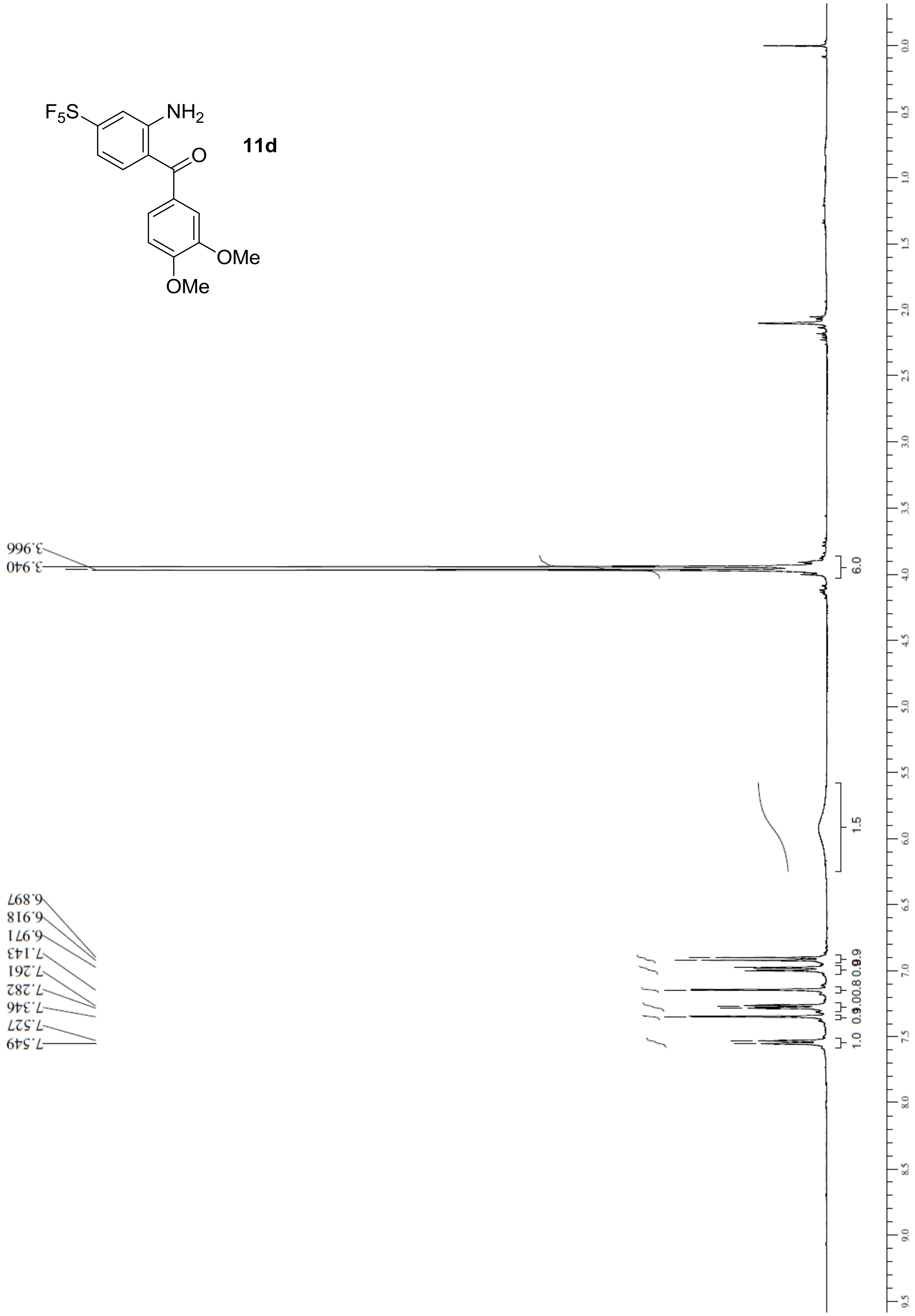

F5S
NH2
O
OMe
OMe
3.966
3.940
6.897
6.918
6.971
7.143
7.261
7.282
7.346
7.527
7.549
6.0
1.5

11d

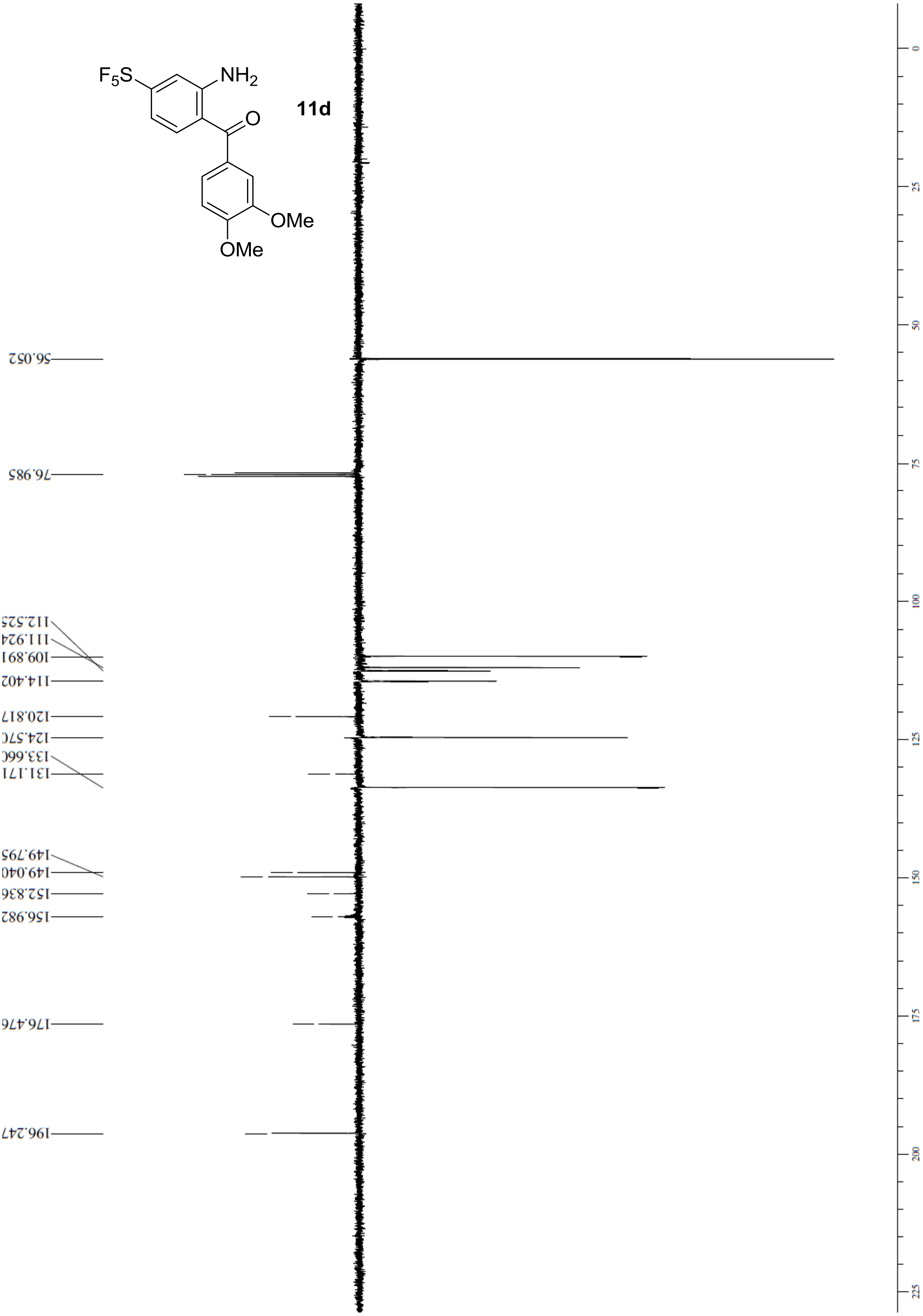
11d
56.052
76.985
112.525
111.924
109.891
114.402
120.817
124.570
133.660
131.171
149.795
149.040
152.836
156.982
176.476
196.247

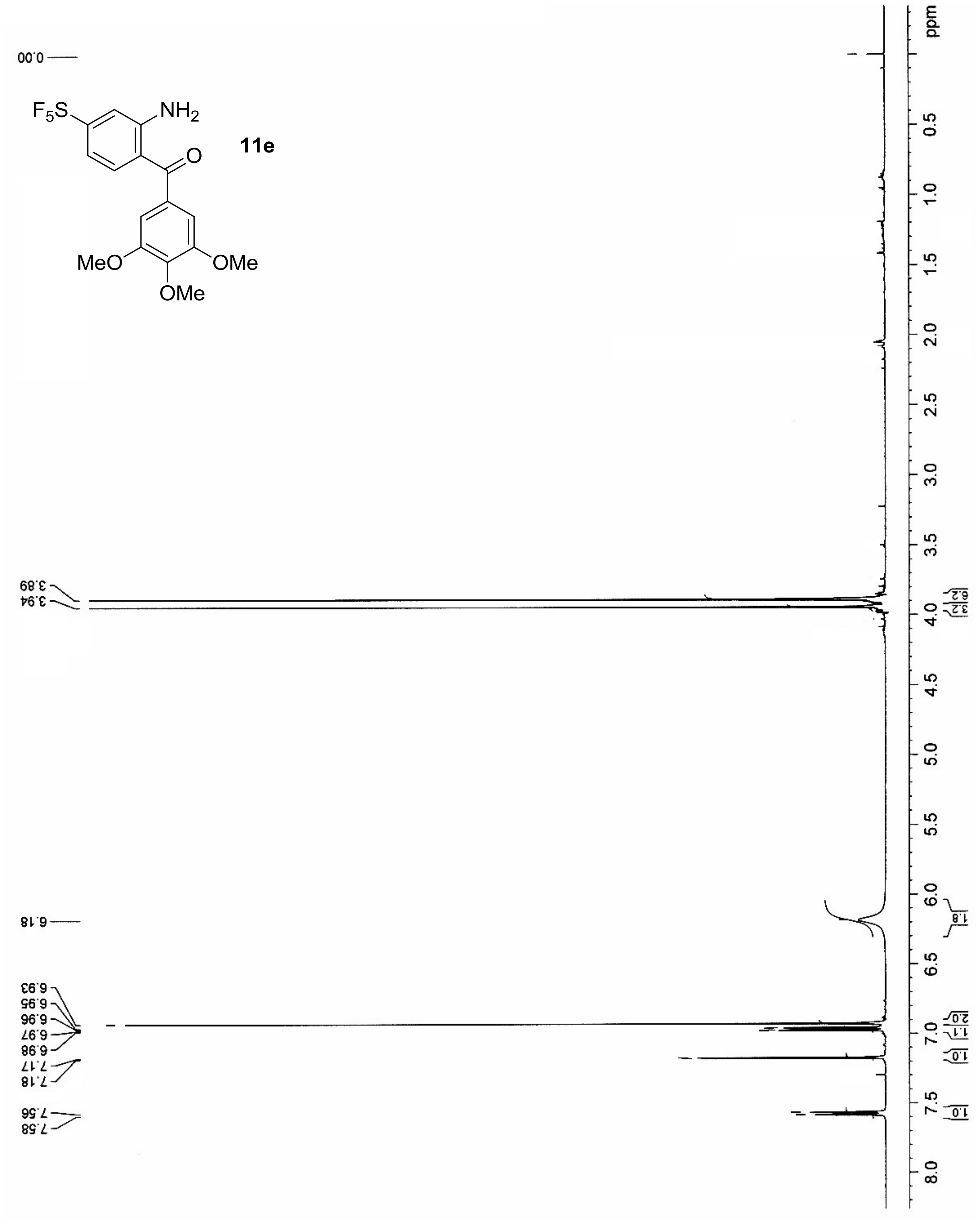
11e
ppm

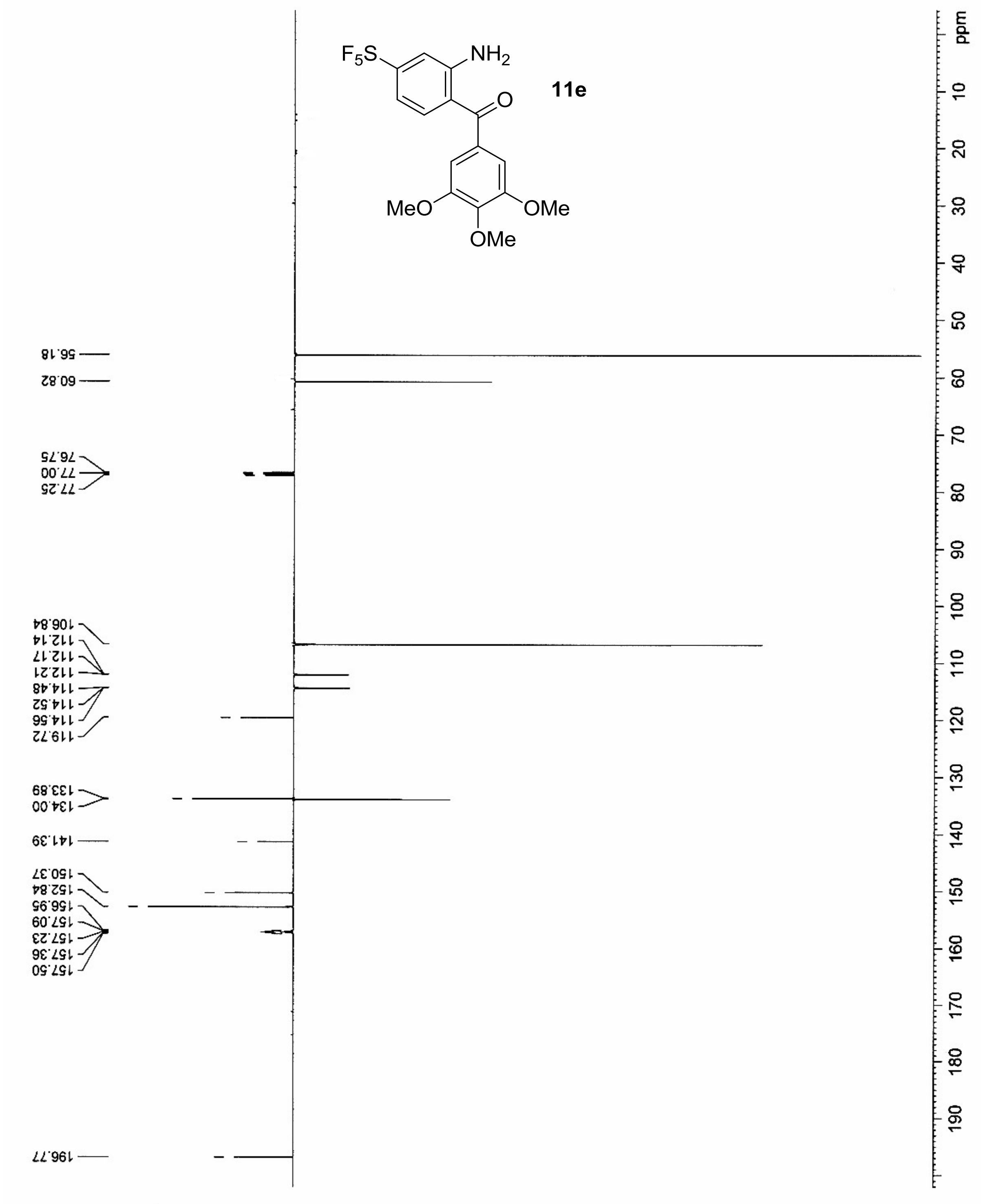

F_5S
NH_2
O
11e
MeO
OMe
OMe
ppm
56.18
60.82
76.75
77.00
77.25
106.84
112.14
112.17
112.21
114.48
114.52
114.56
119.72
133.89
134.00
141.39
150.37
152.84
156.95
157.09
157.23
157.36
157.50
196.77

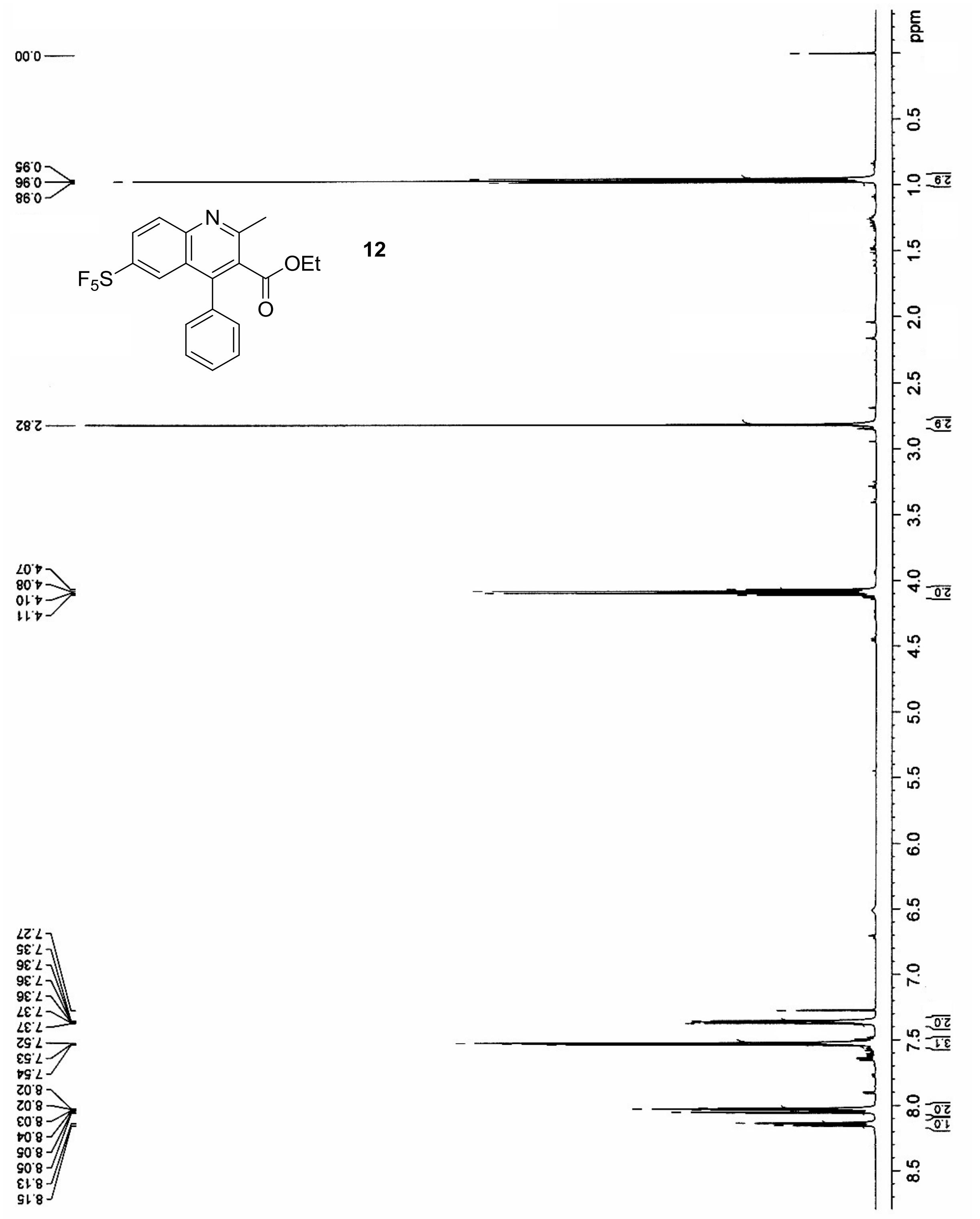
12
ppm
F5S
OEt

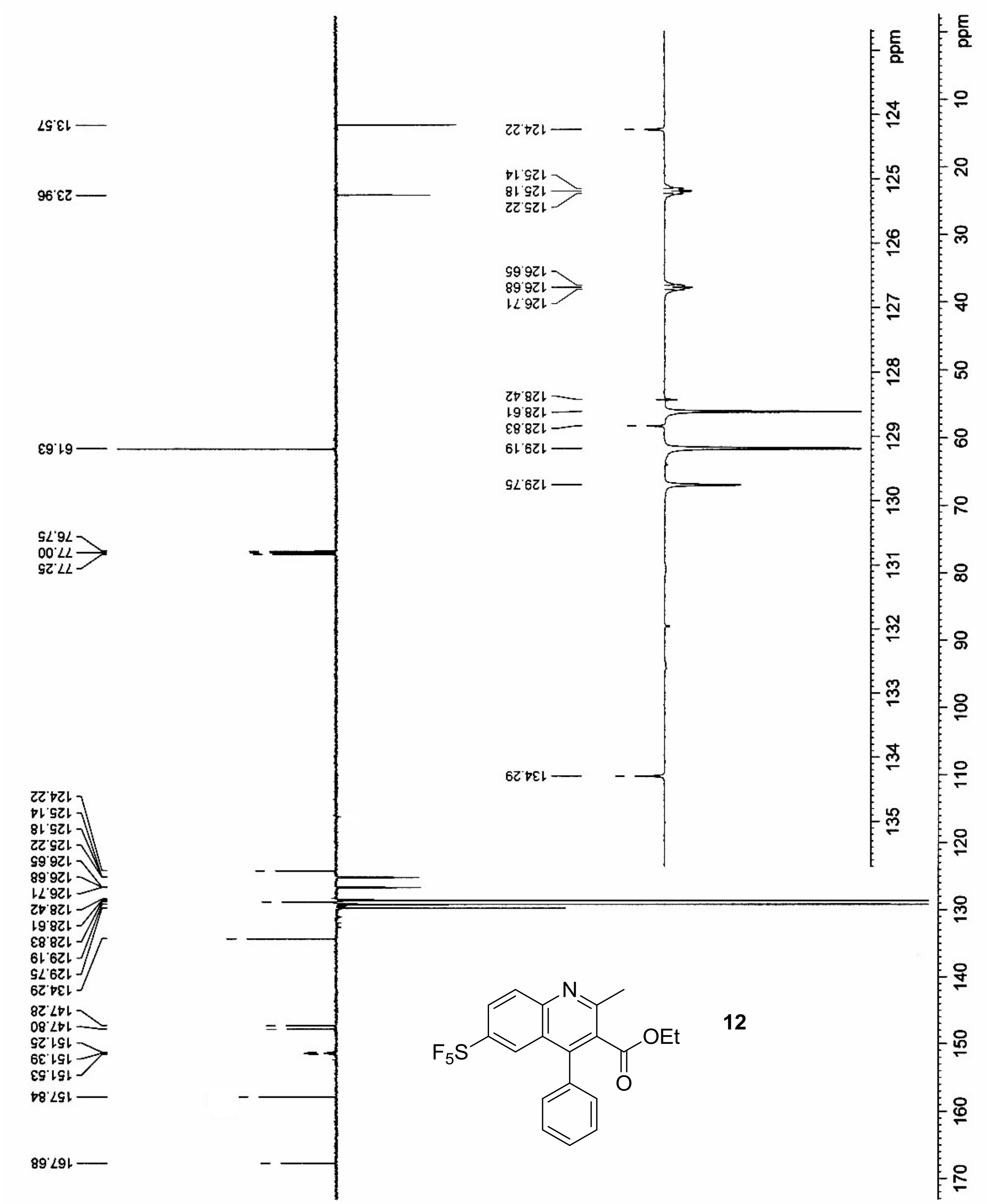
13.57
23.96
61.63
76.75
77.00
77.25
124.22
125.14
125.18
125.22
126.65
126.68
126.71
128.42
128.61
128.83
129.19
129.75
134.29
147.28
147.80
151.25
151.39
151.53
157.84
167.68
ppm
F_5S
N
OEt
O
12

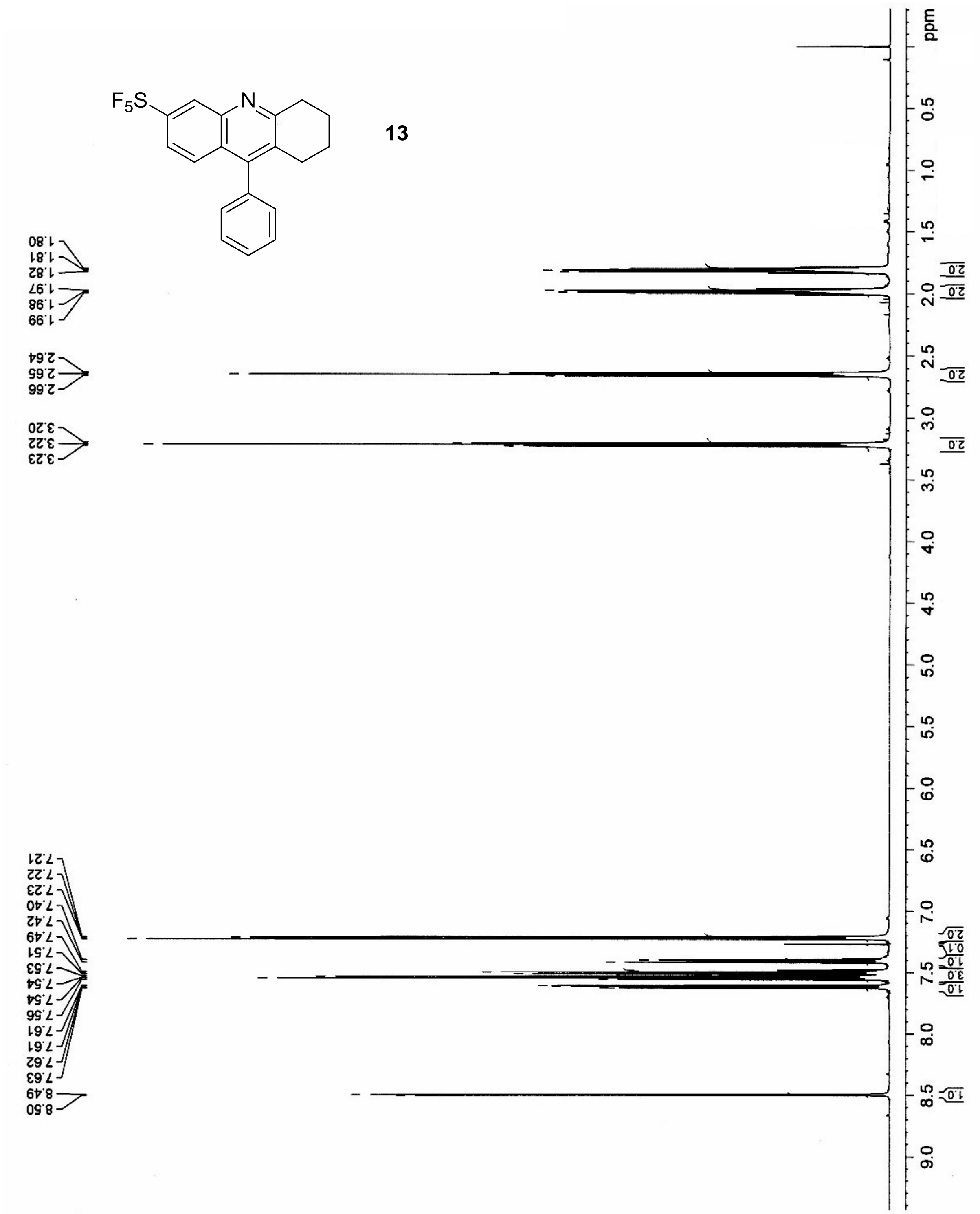
13

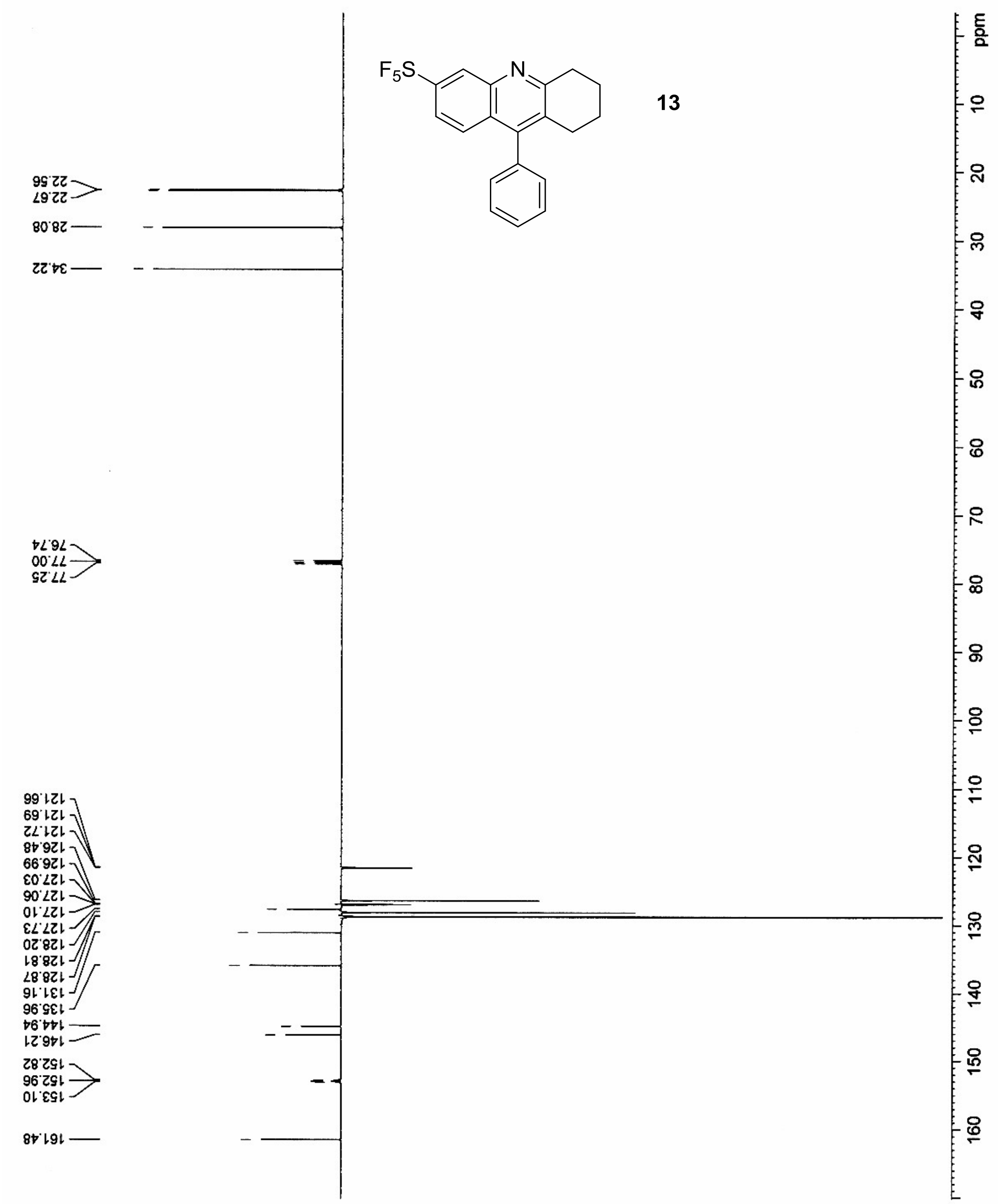
F5S
N
13
22.56
22.67
28.08
34.22
76.74
77.00
77.25
121.66
121.69
121.72
126.48
126.99
127.03
127.06
127.10
127.73
128.20
128.81
128.87
131.16
135.96
144.94
146.21
152.82
152.96
153.10
161.48
ppm
10
20
30
40
50
60
70
80
90
100
110
120
130
140
150
160

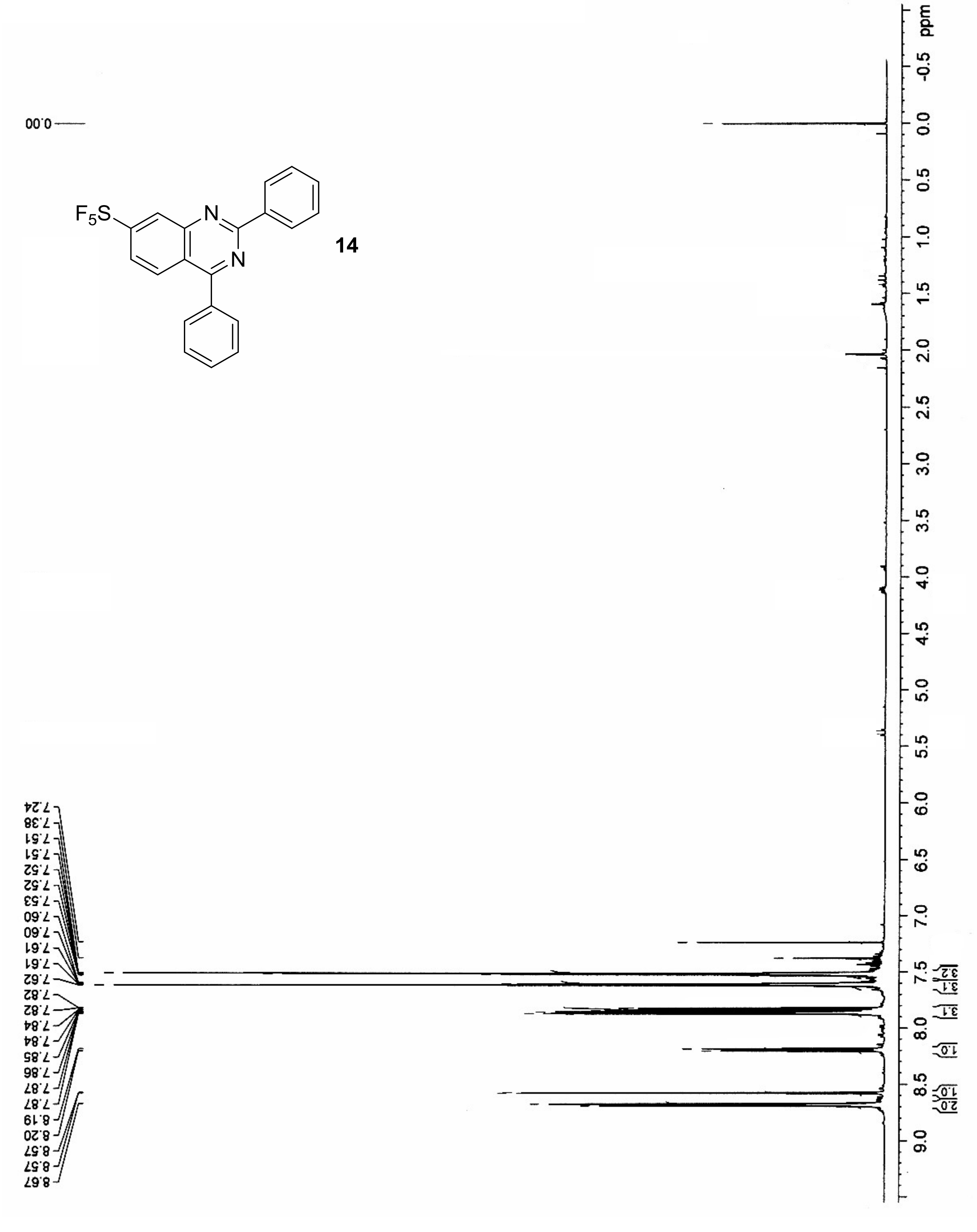

F_5S
N
N
14
0.00
7.24
7.38
7.51
7.51
7.52
7.52
7.53
7.60
7.60
7.61
7.61
7.62
7.82
7.82
7.84
7.84
7.85
7.86
7.87
7.87
8.19
8.20
8.57
8.57
8.67
ppm

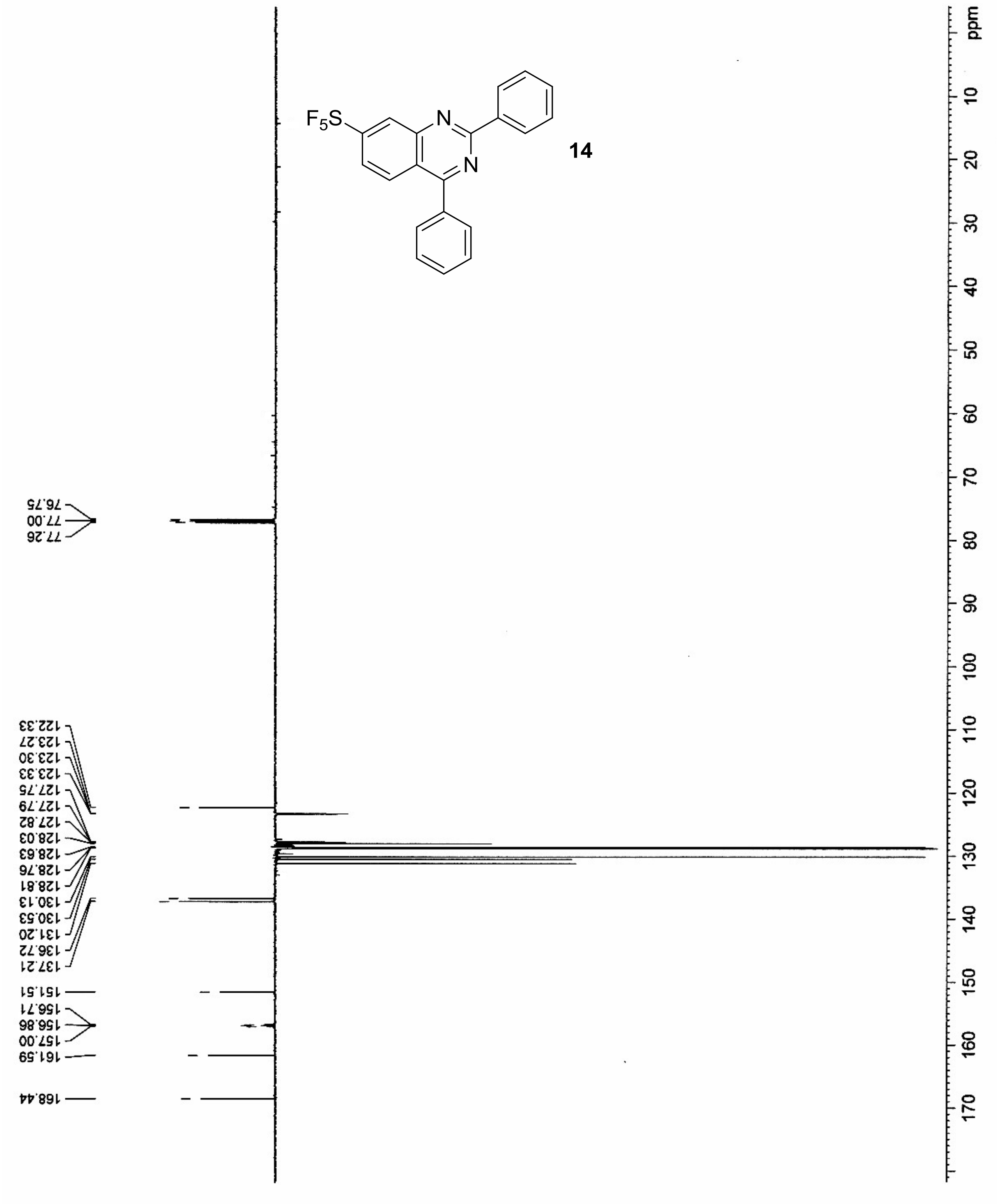

14
76.75
77.00
77.26
122.33
123.27
123.30
123.33
127.75
127.79
127.82
128.03
128.63
128.76
128.81
130.13
130.53
131.20
136.72
137.21
151.51
156.71
156.86
157.00
161.59
168.44
ppm
